# LSTM-Powered COVID-19 prediction in central Thailand incorporating meteorological and particulate matter data with a multi-feature selection approach

**DOI:** 10.1016/j.heliyon.2024.e30319

**Published:** 2024-04-26

**Authors:** Chanidapa Winalai, Suparinthon Anupong, Charin Modchang, Sudarat Chadsuthi

**Affiliations:** a Department of Physics, Faculty of Science, Naresuan University, Phitsanulok 65000, Thailand; b Department of Chemistry, Mahidol Wittayanusorn School (MWIT), Salaya, Nakhon Pathom 73170, Thailand; c Biophysics Group, Department of Physics, Faculty of Science, Mahidol University, Bangkok 10400, Thailand; d Centre of Excellence in Mathematics, CHE, Bangkok 10400, Thailand; e Thailand Center of Excellence in Physics, CHE, 328 Si Ayutthaya Road, Bangkok 10400, Thailand

**Keywords:** Multi-feature selection, COVID-19, Long short-term memory model, Meteorology, Particulate matter

## Abstract

The COVID-19 pandemic has significantly impacted public health and necessitated urgent actions to mitigate its spread. Monitoring and predicting the outbreak's progression have become vital to devise effective strategies and allocate resources efficiently. This study presents a novel approach utilizing Multivariate Long Short-Term Memory (LSTM) to analyze and predict COVID-19 trends in Central Thailand, particularly emphasizing the multi-feature selection process. To consider a comprehensive view of the pandemic's dynamics, our research dataset encompasses epidemiological, meteorological, and particulate matter features, which were gathered from reliable sources. We propose a multi-feature selection technique to identify the most relevant and influential features that significantly impact the spread of COVID-19 in the region to enhance the model's performance. Our results highlight that relative humidity is the key factor driving COVID-19 transmission in Central Thailand. The proposed multi-feature selection technique significantly improves the model's accuracy, ensuring that only the most informative variables contribute to the predictions, avoiding the potential noise or redundancy from less relevant features. The proposed LSTM model demonstrates its capability to forecast COVID-19 cases, facilitating informed decision-making for public health authorities and policymakers.

## 1 Introduction

The COVID-19 pandemic has left an impact on global health and society, affecting every part of the world. As the pandemic continues to evolve, there is growing recognition of the need to understand COVID-19 in the context of endemicity, where the virus becomes established and circulates continuously within a population. The transition from a pandemic to an endemic state poses particular issues in managing the disease's new normal [1]. The negative consequences of the pandemic on normal operations can be examined across various domains. Related studies exploring issues such as the closure of facilities due to COVID-19 lockdowns [2], disruptions in supply chains [[3], [4], [5]], the sudden transition to remote operations [6], adherence to social distancing norms in the post-epidemic period [7], the interplay between trust and risk perception in accepting measures to curb COVID-19 cases [8], and the subsequent challenges in maintaining productivity among employees with elevated risk perceptions of COVID-19 [9].

Meteorological variables such as temperature, humidity, rainfall, and wind speed, along with air quality indicators such as PM2.5 and PM10, have been shown to influence the COVID-19 infection [[10], [11], [12], [13], [14], [15]]. In a study encompassing 20 out of 187 nations, it was observed that elevated temperatures and increased relative humidity are associated with a reduction in the viability, stability, survival, and transmission of COVID-19 [13]. The study examining the impact of weather on SARS-CoV-2 transmission in 500 locations worldwide found that the virus spreads more readily in climates with temperatures ranging from 5 to 15 °C, relative humidity of 70%–80 %, wind speeds of 1.5–4.5 m/s, and when visibility is less than 10 statute miles [10]. Chen et al. found that there is a 5.4 % decrease in COVID-19 cases associated with a 1 °C increase in temperature [11]. A study in China underscores that a decrease in wind speed within the range of 1.5–2.5 m/s corresponds to a reduced risk of COVID-19 spread; conversely, an increase in wind speed heightens the risk [15]. Globally, a comprehensive study highlights the role of rainfall in amplifying the spread of COVID-19, indicating that each 1-inch/day increase leads to an additional 56.01 cases per day [14]. In addition, some studies have directed their attention towards particulate matter, as evidenced by research that sought to examine the pollution levels of particulate matter (PM) and its potential association with the incidence of COVID-19 in four Italian cities: Milan, Rome, Naples, and Salerno [12].

Research on the interaction between COVID-19 and these environmental factors is ongoing, and our understanding of these relationships continues to evolve. Public health authorities consider these factors when developing strategies to control the spread of the virus, especially in regions with extreme weather conditions or significant air quality issues [16].

This study aims to encompass a comprehensive analysis of COVID-19 trends in Central Thailand. First, we aim to conduct a thorough examination of the existing patterns of the disease in the region. Then, we seek to identify and analyze influential factors that significantly contribute to the transmission of COVID-19 using multi-feature selection techniques. Finally, our goal is to develop a predictive model for the COVID-19 progression using data collected over a period of 2 years. Through these objectives, our study desires to contribute valuable insights that can inform evidence-based strategies for managing and mitigating the impact of the pandemic in Central Thailand.

This study sets out to investigate the potential of the Multivariate Long Short-Term Memory predictive model, in conjunction with meteorological and particulate matter factors, employing a comprehensive approach of multi-feature selection with lag time selection through XGBoost, Random Forest (RF), Selection by Filtering (SBF), Principal Component Analysis (PCA), and correlation analysis. To ensure a comprehensive examination of predictive capabilities, we also conduct a comparative analysis to assess the efficacy of the LSTM model. This comparison involves evaluating its performance against both the Recurrent Neural Network (RNN) and the Generalized Linear Model (GLM). Through the integration of these methodologies and a prolonged data collection period, this study can provide significant insights into forecasting and comprehending the dynamics of COVID-19 within Central Thailand.

This paper is organized into six sections. The introduction in section 1 highlights the influence of meteorological and air quality factors on COVID-19 transmission and sets the stage for a comprehensive analysis of COVID-19 trends in Central Thailand. Section 2 provides a literature review covering the COVID-19 situation in Thailand, time series predictive models, and feature selection techniques. Section 3 outlines the methodology, including data collection, preprocessing, multi-feature selection techniques, and the Multivariate Long Short-Term Memory (LSTM) predictive model. Section 4 presents the results of the study, including insights gained from the analysis of COVID-19 trends and the performance of the predictive model. Section 5 discusses the findings, their implications, and avenues for future research. Finally, we concluded our key findings and contributions of the study in section 6.

## 2 Literature review

This section is structured to provide a comprehensive understanding of the research context and the methodologies adopted in previous studies. It commences with subsection 2.1, offering an overview of the COVID-19 situation in Thailand. Subsection 2.2 explores the literature concerning time series predictive models, while subsection 2.3 investigates various feature selection techniques.

### 2.1 COVID-19 situation in Thailand

The emergence of SARS-CoV-2, the virus responsible for COVID-19, originated in Wuhan, China. The first COVID-19 case outside mainland China was identified in Thailand on January 13, 2020 [17]. The number of confirmed COVID-19 cases in Thailand saw a sharp increase following significant outbreaks at Lumphini Boxing Stadium and Thong Lo entertainment venue in early March 2020. In response, the country implemented various measures to control the spread of the virus, including travel restrictions, lockdowns, and social distancing measures in April 2020. The first wave peaked on March 22, 2020, with 3016 cases reported in Thailand [17,18]. Subsequently, a second COVID-19 wave began on December 17, 2020, prompting the government to swiftly implement localized lockdowns and active case detection strategies [19]. The highest number of cases throughout the first two years was 23,418 in August 2021 [18,20,21]. The COVID-19 transmission in Thailand is still active and pervasive today, particularly in the central region of the country [21]. The central area of Thailand has been particularly hard-hit by the virus, recording its peak in August 2021 with 13,352 cases, constituting 57 % of the nationwide cases, as shown in Fig. S1. In regions with fluctuating climates like Central Thailand, it is crucial to investigate the relationship between meteorological and air quality factors and COVID-19 transmission to develop more accurate predictive models.

Our study boards on an analysis of COVID-19 from an endemic viewpoint, delving into the virus's statistical dynamics within a specific population over an extended period. The study, focused on Thailand's initial significant pandemic phase spanning two years, represents a critical juncture wherein the virus becomes ingrained in the community's infectious disease landscape. The central region was deliberately chosen due to significant variations in weather, travel patterns, and behavioral aspects across different regions [22]. Remarkably, the dynamics within this specific region wield considerable influence over the overall trends in the nation. This convergence may be attributed to a combination of factors, including population density, and prevailing social gathering habits within the central region. Such a pivotal role underscores the significance of focusing on the central region in understanding and addressing the broader dynamics of the COVID-19 situation in Thailand. Accordingly, understanding the dynamics of COVID-19 transmission in the central region of Thailand has become essential for effective public health interventions.

### 2.2 Time series prediction models

A recurrent neural network (RNN) is a generalized artificial neural system that is composed of input, hidden, and output layers [23]. RNN typically processes data using short-term memory, but due to a vanishing gradient problem, it cannot process long data sequences [23,24]. To address this issue, Hochreiter & Schmidhuber introduced the Long Short-Term Memory (LSTM), which facilitates the retention of important data from previous stages to the current one [24]. Due to its capacity for long-term information storage, LSTM is frequently used for analyzing and forecasting time-series data [24].

In several studies, 10.13039/100014976LSTM deep learning models have been shown to have an advantage in COVID-19 forecasting compared to other machine learning neural networks, for example, Multilayer Perceptron (MLP) [25], Recurrent Neural Network (RNN) [26], and Support Vector Machine (SVM) [27], as well as statistical approaches such as Autoregressive Integrated Moving Average (ARIMA) [25,28], which exhibited inconsistent performance. A study conducted in New Zealand also supported the effectiveness of 10.13039/100014976LSTM in assessing trends that reduce the number of daily events to zero [29]. However, the hybrid method of the multi-head attention mechanism and the statistical features (ATT_FE) technique is more effective than the LSTM model [30]. Specific conditions may be required for optimal performance of the deep learning models, such as selecting the appropriate time delay. For instance, univariate LSTM models showed good accuracy only for short-term predictions (1-day lead) in certain cases [26,31]. However, the effectiveness of LSTM predictive models can vary based on feature conditions and the choice of time delay, emphasizing the importance of careful model selection based on the specific context and dataset. Thus, in this work, we aim to develop an LSTM model to forecast the progression of the COVID-19 outbreak.

### 2.3 Feature selection

Constructing a predictive model necessitates choosing suitable techniques that are aligned with the data, including being able to select important features, lag times, and data size [32]. The selection of lag time in a predictive model is crucial for achieving accurate and reliable predictions [33]. Lag time refers to the time delay between the input variables and their impact on the target variable. By carefully choosing the appropriate lag time, we can ensure that the model effectively incorporates the relevant historical information to make predictions [32].

Feature selection involves identifying the most relevant and informative input variables that contribute significantly to the target variable's prediction. Including irrelevant or redundant features may introduce noise and lead to overfitting, reducing the model's generalization ability. Therefore, selecting the most important and meaningful features is essential for enhancing the model's performance and interpretability [33,34]. Various techniques have been developed to accomplish feature selection. In general, there are three methods: filter methods, wrapper methods, and embedded methods.

Filter methods assess the relevance of features independently of the chosen model and utilize statistical measures, such as correlation coefficients or mutual information, to rank and select features [35,36]. Filter methods are recognized for their simplicity and efficiency. Filter methods are less computationally demanded than wrapper methods, making them particularly suitable for high-dimensional datasets. Despite their advantages, filter methods may overlook intricate feature interactions specific to certain models [37]. Nevertheless, their simplicity and speed make them a popular choice, especially in scenarios where computational resources are limited and a quick initial feature screening is desirable [[35], [36], [37]].

One of the famous methods for filter methods is XGBoost. Numerous studies have employed XGBoost as a valuable tool to decide significant factors in relation to COVID-19, encompassing aspects such as population dynamics, vaccine-related variables, and meteorological conditions. The outcomes consistently demonstrate that XGBoost plays a pivotal role in identifying influential factors that aid in feature selection, contributing to the improved understanding of the pandemic's complexities [38].

The feature importance in XGBoost was automatically computed to find the contribution of each attribute split point and enhance the performance measure, which was weighed based on the number of associated observations. This weighted contribution was then used to assess the importance of each attribute split point in a single decision tree. Additionally, the feature significance scores provided relative weights for different features, indicating their importance in the analysis [38,39].

Wrapper methods for feature selection have gained attention as an effective strategy to optimize machine learning model performance. It involves evaluating feature subsets through trial and error, using a specific model's performance as a criterion for selection. These methods directly integrate the machine learning model into the feature selection process, modifying the selection criteria to the model's intricacies. Their iterative nature allows continuous refinement of feature subsets, promoting stability in selected features. In general, wrapper methods offer a robust and customized approach to feature selection, leading to enhanced model generalization and improved performance across various machine learning applications [37].

Wrapper methods have gained attention in COVID-19 research as a powerful approach for selecting relevant features and optimizing predictive models. Several studies have explored the application of wrapper methods in the context of COVID-19 analysis [[40], [41], [42], [43]]. Researchers have utilized techniques like Recursive Feature Elimination (RFE), Genetic Algorithms (GA), Selection by Filtering (SBF), and Sequential Feature Selection (SFS) to identify factors such as demographic information, comorbidities, and environmental variables that significantly impact COVID-19 outcomes. By selecting the most informative features, these studies aim to enhance the accuracy of predictive models [40,41].

Selection By Filtering (SBF) was applied in the various research that constructs the model fit after employing univariate filters. In feature selection for the training data, this method allows for obtaining resampling estimates for the models. In each resampling iteration, the predictor variables undergo univariate filtering before modeling. The performance of this technique is assessed using a resampling [42,43].

Embedded methods integrate feature selection within the model-building process. These methods optimize feature relevance alongside model parameters, often leading to more efficient and accurate models [40]. Embedded methods are advantageous as they streamline the feature selection process, eliminating the need for a separate step, and they tend to capture intricate relationships between features and the target variable [40].

The Random Forest is a powerful ensemble-learning algorithm that constructs numerous simple classifiers using randomly selected features, ensuring comprehensive exploration of potential feature subsets. Bootstrap Aggregating (Bagging) is a technique used in the Random Forest algorithm. In bagging, multiple subsets of the original dataset are produced using replacement sampling and random sampling. Then, a different decision tree is trained using each subset individually. Bagging is a crucial component of this method, and not all training data is utilized to create each base hypothesis [44,45]. The ensemble is composed of classification and regression decision trees, where the regression tree applies a series of hierarchically ordered conditions from the root to the leaf of the tree [46]. To evaluate feature subsets, the out-of-bag data can be used without requiring the test set. Due to these characteristics, Random Forest proves to be a promising choice for feature selection in the analysis [45]. COVID-19 studies have also employed Random Forest (RF) feature selection to enhance the accuracy of predictive models. By ranking variables based on their importance scores within the ensemble of decision trees, Random Forest identifies the most influential features, improving the model's capability to predict outcomes and disease trends very well [47,48].

The realm of feature selection techniques provides a wide range of techniques to improve model performance, increase effectiveness, and enable insights into complicated datasets. The choice of technique depends on the dataset, the modeling goal, and the balance between interpretability and predictive power. Several studies have focused on developing COVID-19 prediction models. However, research specific to the COVID-19 pandemic in Thailand has been relatively limited [49], and as far as we know, none have used such a long period (2 years) to predict the COVID-19 situation.

## 3 Methodology

The methodology section is organized into subsections for clarity and coherence. 3.1, 3.2 provide the details on the collection and preprocessing of the data. Subsection 3.3 explains the process of identifying relevant features and lag times using the multi-feature selection technique. Subsection 3.4 describes the Long Short-Term Memory (LSTM) model, providing insights into LSTM network construction and comparisons with other models. Subsection 3.5 shows the performance metrics, and subsection 3.6 provides the details on the software used for the analysis.

### 3.1 Data collection

COVID-19 case data [50], along with meteorological and air quality factors, were gathered from 18 provinces within Thailand's central region, including Bangkok, Samut Prakan, Samut Sakhon, Samut Songkhram, Nakhon Pathom, Nonthaburi, Pathum Thani, Nakhon Sawan, Phra Nakhon Si Ayutthaya, Ang Thong, Singburi, Uthai Thani, Chainat, Lopburi, Suphanburi, Kanchanaburi, Saraburi, and Ratchaburi based on data from the Thai Meteorological Department [51]. The study period ranged from January 1, 2020, to December 31, 2021.

The particulate matter data were sourced from the World Air Quality Index (WAQI) [52], which provides the PM2.5 and PM10 concentrations. The meteorological variables were gathered from the Global Surface Summary of the Day (GSOD) [53], which provides the daily meteorological variables encompassing temperature, rainfall, relative humidity, wind speed, visibility, and standard pressure. The details and sources of data are shown in Table 1. The COVID-19 reported cases were obtained from the Open Government Data. The raw plot depicting meteorological, air quality factors, and COVID-19 reported cases data within that timeframe can be observed in Supplementary Fig. S2.

**Table 1:** Descriptions of the parameters.

Parameters	Descriptions	References
Cases	COVID-19 reported cases in the central region, Thailand (cases)	[50]
PM2.5	Mean Particulate Matter 2.5 concentration; PM2.5 (μg/m^3^)	[52]
PM10	Mean Particulate Matter 10 concentration; PM10 (μg/m^3^)	[52]
Temperature	Mean temperature (Celcius)	[53]
Rainfall	Mean precipitation (mm)	[53]
Relative Humidity	Mean relative humidity; RH (%)	[53]
Wind Speed	Mean wind speed (m/s)	[53]
Visibility	Mean visibility (km)	[53]
STP	Mean standard pressure of the day (hPa)	[53]

### 3.2 Data preprocessing

**Fig. 1:**
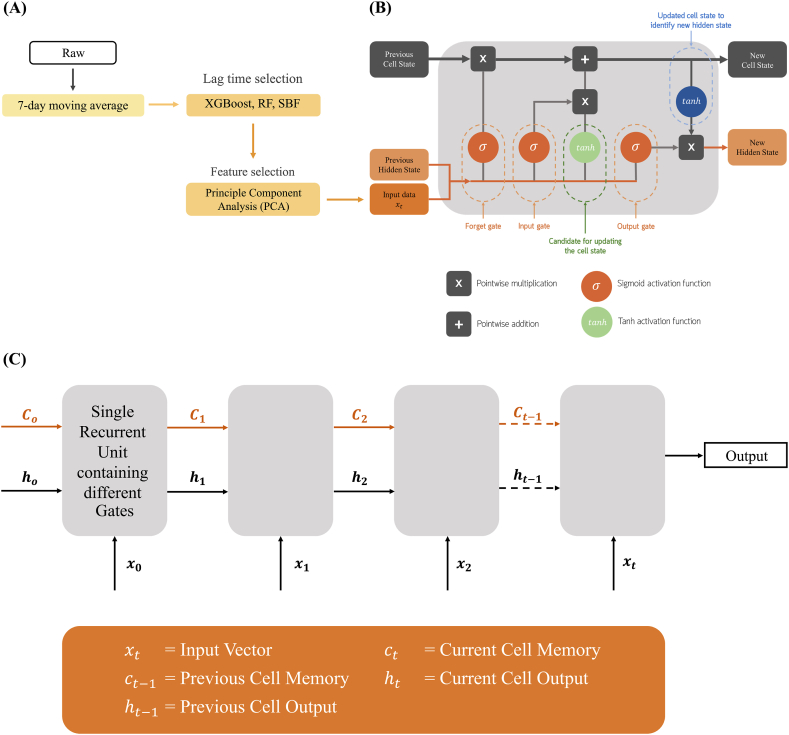
Detailed Structure of an LSTM Memory Cell in an LSTM Recurrent Neural Network. (A) Data preprocessing, lag time selection, and feature selection, (B) An LSTM unit cell architecture, and (C) LSTM model.

Data preprocessing is a crucial step in preparing raw data for analysis and modeling. It involves cleaning, transforming, and organizing data for tasks like machine learning and statistical analysis. We handled the missing data using linear interpolation. As our focus was on reported cases within Central Thailand, comprising 18 provinces, we summed the total COVID-19 cases. For meteorological and air quality factors, the data were averaged over 20 stations and 24 stations, respectively. All features, including COVID-19 cases, were smoothed using a 7-day moving average and then normalized for applying to the multivariate LSTM model (Fig. 1(A)). The input data for feature selection and the model are provided in supplementary file 2.

### 3.3 Multi-feature selection technique

Feature selection techniques are necessary to investigate the association between features and the number of cases used to create a predictive model. A multi-feature selection technique is proposed to choose a subset of variables. We started with lag selection to determine the most suitable lag time for each factor relative to COVID-19 cases. In this work, we used a variety of feature selection techniques, proposed to be a multi-feature selection technique as described in the literature review section. Specifically, we selected three alternative techniques, each representing a different category of feature selection approaches. From the filter method, we selected the XGBoost as our chosen technique. For the embedded method, we employed Random Forest (RF). Finally, we used Selection By Filtering (SBF) for the wrapper method. We, thus, applied XGBoost, RF, and SBF to select lag times for each variable where the maximum lag time is equal to 21 days according to an isolation duration guideline [54]. From these three approaches, we choose the overlapping lag for each variable that aligns with the top four rankings across the methods, ensuring consistency in lag selection.

After implementing the lag-time selection approach, we identified the selected variables along with their respective lag times. To further refine the analysis, we find the factors that exhibit the strongest correlation with the number of COVID-19 cases and avoid interdependencies among input variables. We first applied variable clustering by employing Principal Component Analysis (PCA). The primary objective of PCA is to minimize the interconnections between variables while retaining the maximum data variance [55,56], where the outcome of PCA is presented in a biplot, illustrating the variable clusters. Then, we considered the correlations between each variable and the number of cases for each cluster. From each cluster, we choose a single variable based on the highest correlation coefficients, calculated using equation (1).

(1) $$corr=\frac{\sum \left(x_{i}-\;\bar{x}\right)\left(y_{i}-\;\bar{y}\right)}{\sqrt{\sum {\left(x_{i}-\;\bar{x}\right)}^{2}{\left(y_{i}-\;\bar{y}\right)}^{2}}}\text{,}$$

where $x_{i},y_{i}$ are the weather variable data and the number of COVID-19 cases, respectively,

$\bar{x},\bar{y}$ are the mean of weather variable data and the number of COVID-19 cases, respectively.

### 3.4 Long short-term memory (LSTM) model

The chosen variables serve as the input features for constructing the prediction models. Long Short-Term Memory (LSTM) networks belong to the category of recurrent neural networks, enabling them to grasp sequential dependencies in prediction tasks [57]. Data will be divided into batches. In one training iteration, LSTM batch size indicates how many input sequences are processed together. The model weights are updated after each batch. Larger batch sizes can speed up training but demand more memory.

Comprising an input layer, one or more hidden layers, and an output layer, LSTM can effectively retain essential information from earlier sequences and carry it forward. Within an LSTM layer, we have nodes, also known as memory cells. These cells signify the individual computational units responsible for preserving and handling information over a span of time. Nodes in an LSTM store information in memory and control how information is passed through the sequence of data. With the inclusion of a hidden state, LSTM's processing of recurrent units becomes significantly more sophisticated than traditional RNNs [23]. The process of LSTM's recurrent unit is depicted in Fig. 1(B).

In the LSTM model, the input features comprise a sequence of input vectors denoted as (x₁,x₂,x₃,...,xₜ), where t represents the time step. Before being processed through the forget gate, the current step's input (xₜ) and the previous step's hidden state (hₜ₋₁) are combined. The forget gate, utilizing the sigmoid function, determines which information to discard, ranging between 0 and 1, thereby deciding which data from the previous hidden state should be retained. Simultaneously, the input gate determines which components of the current input (xₜ) should be included in the cell state (cₜ). The input gate also considers the previous hidden state (hₜ₋₁). The cell state (cₜ) is updated based on the information passed through both the forget gate and the input gate. By storing relevant information from past time steps, the LSTM can effectively maintain long-term dependencies. Additionally, the output gate considers both the updated cell state and the current input (xₜ) to determine which parts of the cell state will be utilized in generating the current hidden state (hₜ). The LSTM model produces the current hidden state (hₜ), capturing pertinent information from the current input and previous time steps. This hidden state serves as the output and is subsequently used for further predictions or passed to subsequent time steps in the sequence as indicated in Fig. 1(C).

One epoch refers to a complete pass through the entire training dataset during the training process. During each epoch, the LSTM model updates its weights and biases based on the training data to minimize the error and improve its performance in predicting the target output. The LSTM model was calculated in order, as shown in Table 2, where $i_{t}$, $f_{t,}$ and $o_{t}$ refer to the gates of input, forget and output at time t, respectively. $x_{t}$ and hₜ are the number of input features and the number of hidden units, respectively. W is the weight matrices, and b is the bias [23,24].

**Table 2:** LSTM unit formulas at the time step, t:

Component	Formula
Input Gate	$i_{t}=\sigma \left(W_{i}\left[h_{t-1},x_{t}\right]+b_{i}\right)$
Forget Gate	$f_{t}=\sigma \left(W_{f}\left[h_{t-1},x_{t}\right]+b_{f}\right)$
Cell Candidate	$g_{t}=\tanh\left(W_{g}\left[h_{t-1},x_{t}\right]+b_{g}\right)$
Output Gate	$o_{t}=\sigma \left(W_{o}\left[h_{t-1},x_{t}\right]+b_{o}\right)$

The multivariate LSTM models were constructed using the Python language, involving 1000 epochs with two layers. Both two layers of LSTM consist of 8 units and the dense layer consists of 32 unit with optimizer “adam” and batch size set to 10. This refinement led to improved accuracy in the results. To ensure the reliability of our models, we performed 100 experiments and calculated the Mean Absolute Error (MAE) and Root Mean Square Error (RMSE) to identify the most optimal prediction model.

This study also compares the efficiency of the Long Short-Term Memory (LSTM) model against the Recurrent Neural Network (RNN) and Generalized Linear Model (GLM) to ensure a comprehensive evaluation. Through the integration of diverse machine learning and statistical techniques, the study provides a comparison of error values for each model concerning the study's objectives. Additional details regarding the methodologies employed for RNN and GLM are available in the supplementary.

### 3.5 Performance metrics

We combined the selected parameters from feature selection and the number of cases to be our input parameters, which were used to construct multivariate LSTM models. Training and testing datasets were designed, representing 85 % and 15 % of the total data, respectively. The model performances were assessed using the Mean Absolute Error (MAE) metric (Equation (2)) and the Root Mean Square Error (RMSE) metric (Equation (3)).

(2) $$MAE=\sum_{i=1}^{n}\;\frac{\left|\hat{y_{i}}-y_{i}\right|}{n}\text{,}$$

(3) $$RMSE=\sqrt{\sum_{i=1}^{n}\;\frac{{\left(y_{i}-\;\hat{y_{i}}\right)}^{2}\;}{n}}$$

where $\hat{y_{i}}$ is the predicted data,

$y_{i}$ is the observed data,

n is the length of the observed data.

### 3.6 Software

The selection of lag time using XGBoost was automated through the utilization of the “xgb.importance” function within the R package “xgboost”, specifically version 1.7.3.1 [39]. Random Forest decision tree was implemented via “randomForest” version 4.7–1.1 in R packages [58]. SBF scores were computed by the “sbfControl” function in the "caret" package version 6.0–93 [59]. All the plots were generated using the R package “ggplot2” version 3.4.1 [60]. PCA plots were also constructed using the “autoplot” function within the R package “ggplot2” and the correlation plot was plotted using the R package “corrplot” version 0.92 [61]. The development of the proposed LSTM model was carried out using open-source libraries, including Numpy, Pandas, Sklearn, Tensorflow, and Keras in Python.

## 4 Results

The results section is organized to provide a thorough examination of our research findings on the interplay between meteorological variables, particulate matter, and COVID-19 trends in Central Thailand. This section is segmented into two primary subsections. Subsection 4.1 presents the outcomes of our predictive model's multi-feature selection technique, highlighting both its efficiency and accuracy. Subsection 4.2 provides the results of the multivariate LSTM model analysis, emphasizing its predictive capabilities regarding COVID-19 cases along with assessments of its performance.

### 4.1 Multi-feature selection technique

The multi-feature selection technique employed in this study aimed to enhance the efficiency and accuracy of the predictive models for COVID-19 outbreaks by choosing the most relevant features and appropriate lag times. To select lag times and features, this study utilized three distinct methods: XGBoost, RF (Random Forest), and SBF (Selection By Filtering), along with Principle Component Analysis (PCA) and correlation.

We first applied XGBoost, RF, and SBF methods to rank the correlation between the lag time of each feature and COVID-19 reported cases, with a maximum lag time of 21 days. Each method played a crucial role in assessing the importance of the meteorological and particulate matter factors and their relationship with COVID-19 cases. The results of the important rank of 8 variables from XGBoost, RF, and SBF methods are shown in Figs. S3–S10. In this work, we found that XGBoost's importance ranking exhibited the clearest differentiation among each lag. XGBoost's superior ability to handle complex data relationships and its built-in feature selection mechanism makes it a more effective choice for achieving a clearer cluster solution compared to RF and SBF.

To ensure a robust selection of input parameters, the lag time of each variable was determined by considering lag times that are within the top four ranks across all three methods. After implementing the lag-time selection approach, we have identified 14 variables (Table 3).

**Table 3:** Lag time selection of each parameter.

Parameter	Lag time
Temperature	0-day lag and 21-day lag
Rainfall	21-day lag
Relative Humidity	0-day lag
Wind Speed	0-day lag and 21-day lag
Visibility	0-day lag, 20-day lag and 21-day lag
STP	18-day lag
PM2.5	0-day lag and 21-day lag
PM10	0-day lag and 21-day lag

**Fig. 2:**
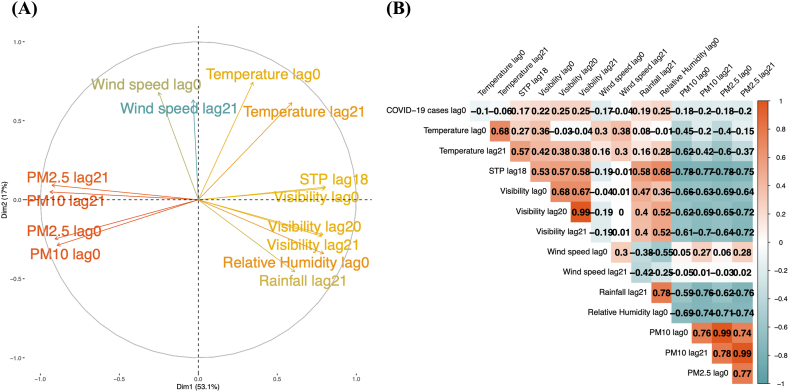
Dimensionality visualization of meteorological and air quality factors data using (A) Principal Component Analysis (PCA) and (B) correlation matrix, which shows the relationships between meteorological, particulate matter factors, and COVID-19 cases. PCA indicates four distinct; Group 1: PM2.5 0-day lag, PM2.5 21-day lag, PM10 0-day lag, and PM10 21-day lag, Group 2: wind speed 0-day lag, wind speed 21-day lag, temperature 0-day lag, and temperature 21-day lag, Group 3: visibility 20-day lag, visibility 21-day lag, relative humidity 0-day lag, and rainfall 21-day lag, and Group 4: standard pressure (STP) 18-day lag and visibility 0-day lag.

After screening the lag time of each factor, we used the Principle Component Analysis (PCA) method to cluster the selected 14 features. The PCA results are presented in Fig. 2(A), demonstrating that the input features are clearly divided into four groups. To avoid self-relation between input parameters, we kept one parameter from each group based on its correlation with the number of COVID-19 cases (Fig. 2(B)).

Among Group 1 in Fig. 2(A), PM10 concentration with a lag of 21 days emerged as the most influential parameter, demonstrating a strong correlation with the number of cases. For Groups 2 and 3, relative humidity and wind speed with no lag were identified as significant predictors. For the remaining group 4, considering the critical importance of STP 18-day lag and visibility 0-day lag, we chose visibility 0-day lag as an input feature in our analysis. Through the feature selection process employing PCA and correlation analysis, we can identify the following key input parameters: relative humidity with no lag (0-day lag), wind speed with no lag (0-day lag), visibility with a 0-day lag, and PM10 with a 21-day lag.

These selected parameters will be used as the inputs in our LSTM prediction models, ensuring the model will be an accurate approach to forecasting COVID-19 dynamics.

### 4.2 Multivariate long short-term memory model

Using the features from the multi-feature selection technique, we further predicted COVID-19 cases in Central Thailand using multivariate long short-term memory (LSTM) in Central Thailand. The data were split into training (85 %) and testing (15 %) datasets. In our analysis, we also conducted a comparison between the Long Short-Term Memory (LSTM) model, Recurrent Neural Network (RNN), and Generalized Linear Model (GLM). The Mean Absolute Error (MAE) and Root Mean Square Error (RMSE) results for these three models are presented in Tables S1 and S2 in the supplementary. Table S1 corresponds to the training dataset, while S2 corresponds to the testing dataset. We found that for all models (A-F), the LSTM model demonstrates the best reduction in error. For the LSTM model, Table 4 displays the results of MAE, RMSE, and standard deviation of 100 experiments, showing that models incorporating either relative humidity (model B) or wind speed (model C) outperform model A, which relies solely on COVID-19 cases. This improvement is evident in the reduction of MAE and RMSE for both training and testing datasets compared to model A. The inclusion of meteorological and particulate matter data enhances the prediction by providing crucial contextual information. Univariate models that rely on COVID-19 case data cannot account for external influences or contextual fluctuations caused by meteorological and air quality factors. This limitation can result in less accurate predictions, especially in situations where weather conditions influence the virus's dynamics. However, using other features such as visibility, PM10, or all of the selected features (model F) including Cases (1-day lag), the model did not show the outperformed due to unrelated features introducing noise into the model.

**Table 4:** Mean Absolute Error (MAE) and Root Mean Square Error (RMSE) of training and testing datasets for multivariate LSTM models.

Model	Input features	Training		Testing	
		MAE	RMSE	MAE	RMSE
**A**	Cases (1-day lag)	21.18	58.61	65.90	100.21
**B**	Relative Humidity (0-day lag), Cases (1-day lag)	19.49	53.30	58.14	79.87
**C**	Wind speed (0-day lag), Cases (1-day lag)	20.00	53.80	63.55	96.77
**D**	Visibility (0-day lag), Cases (1-day lag)	23.16	67.31	70.53	109.71
**E**	PM10 (21-day lag), Cases (1-day lag)	21.81	60.42	66.10	98.60
**F**	Relative Humidity (0-day lag), Wind speed (0-day lag), Visibility (0-day lag), PM10 (21-day lag), Cases (1-day lag)	25.05	73.37	73.93	123.26

**Fig. 3:**
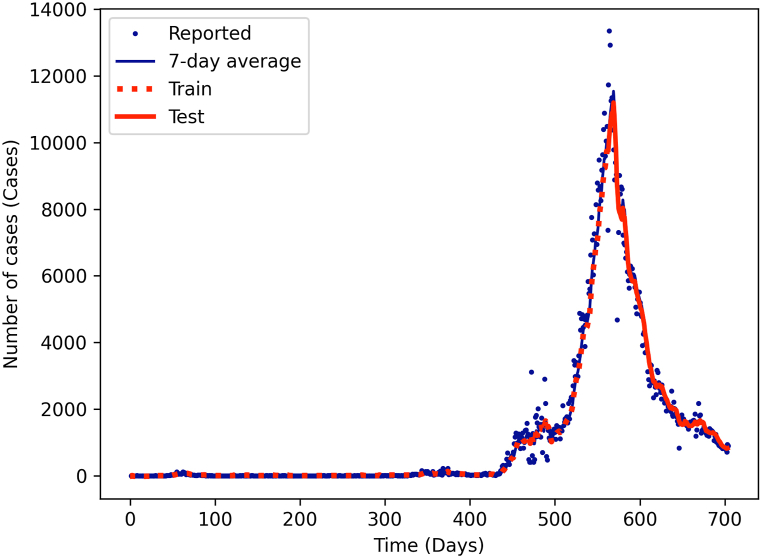
The training (red dashed line) and testing (red solid line) results of model B, comparing with COVID-19 reported cases (blue dots) and their 7-day moving average (blue solid line). (For interpretation of the references to colour in this figure legend, the reader is referred to the Web version of this article.)

All three methods exhibited a consistent trend of results (Tables S1 and S2). Relative humidity consistently demonstrated the best performance in predicting COVID-19 cases in both the training and testing datasets (model B). This result is further supported by the high correlation coefficient observed between relative humidity and the number of COVID-19 cases (Fig. 2(B)). The performance of all the models is presented in the supplementary section Fig. S11. The result of the highest performance model, including relative humidity as one of the input parameters, is displayed in Fig. 3.

## 5 Discussion

This section presents a comprehensive discussion of our research findings, structured into three subsections. Firstly, in subsection 5.1, we delve into our multi-feature selection technique, which aids in determining appropriate lag times and correlations among different factors impacting reported COVID-19 cases. Subsequently, in subsection 5.2, we explore the implementation of a multivariate Long Short-Term Memory (LSTM) model. Finally, in subsection 5.3, we address limitations and provide valuable managerial insights.

### 5.1 Multi-feature selection technique

In this work, we employed advanced methodologies, including XGBoost, Random Forest (RF), and Selection by Filtering (SBF), to assess the correlation between the lag time of each factor and reported COVID-19 cases. The comprehensive application of these methods allowed us to discern the relationship between various factors and the progression of COVID-19. The analysis outcomes show that XGBoost exhibited the clearest differentiation among the lags. This can be attributed to XGBoost's intrinsic feature selection capabilities and its ability to effectively manage complex data relationships.

Gradient boosting from XGboost improves the model's performance by repeatedly including decision trees while concentrating on the most useful characteristics. This process instantly identifies and gives importance scores to features based on their contribution to the model's performance [39,62]. On the other hand, RF also uses decision trees but combines their outputs through averaging or voting [46]. While it's robust and provides feature importance, it might not capture intricate relationships in the data as effectively as XGBoost. Similar to SBF, a wrapper method explores feature subsets to evaluate their impact on model performance [37]. However, this approach can be computationally demanding and may not consistently provide a clear feature separation, particularly in large or complex datasets.

Our feature selection approach is designed to discern and identify the most pivotal factors influencing the dynamics of COVID-19. Through the implementation of a selection process, our objective is to narrow down variables that significantly contribute to the transmission and progression of the disease within Central Thailand. This feature selection methodology not only enhances the precision of our analyses but also enables us to focus specifically on the most relevant and influential factors.

Interestingly, most of the meteorological and particulate matter factors with lag times of 0 and 21 days (except STP and visibility) have been found to have a crucial effect on COVID-19 cases due to their influence on the transmission and survival of the virus. Temperature, relative humidity, wind speed, PM2.5, and PM10, with a lag time of 0 days, directly impact the immediate spread of the virus. Relative humidity and wind speed increase the stability and dispersion of respiratory droplets containing the virus. Higher humidity levels can slow down droplet evaporation, reducing their transmission distance and potentially limiting the spread of the virus [13]. The wind speed decreases the dispersion and dilution of viral particles in the air, influencing the chances of exposure and infection [63].

Factors such as temperature, rainfall, relative humidity, PM2.5, and PM10, with a lag time of 21 days, can influence the virus's incubation period, which is the time it takes for individuals to develop symptoms and seek medical attention [54,64]. These factors can affect the immune response and the virus's ability to survive on surfaces and in the environment. Warmer temperatures, for example, might enhance viral decay and reduce the virus's viability, potentially leading to lower infection rates [65]. Examining these meteorological and particulate matter factors at different lag times provides valuable insights into how environmental conditions impact the transmission and dynamics of COVID-19.

Following a screening of the lag time associated with each feature, we proceeded to employ the Principle Component Analysis (PCA) method to cluster the 14 selected features. The application of PCA offers a comprehensive insight into the inherent relationships and patterns among the variables. The outcomes of this analysis reveal a distinct and meaningful division of the input features into four clearly defined groups. To mitigate self-relations among input parameters, a selection process was applied, wherein one parameter was selected from each clustered group. This selection was made based on the parameter's correlation with the number of reported COVID-19 cases.

The correlation coefficients have revealed two interesting results. Firstly, the correlation between temperature and COVID-19 cases is notably weak. However, in other countries, temperature influences the number of COVID-19 cases [66,67]. The geographical location of each country can contribute to these variations. In the central region of Thailand, the yearly average temperature ranges from 28 to 34 °C, and the number of daylight hours is approximately 11 h. These conditions lead to minimal day-to-day variations compared to other countries. As a result, certain parameters may not significantly impact COVID-19 cases in Thailand.

Another interesting correlation result is that relative humidity exhibits the highest correlation coefficient with COVID-19 cases, corresponding to the best predictive model. This can be attributed to the fact that the transmission rate of COVID-19 is influenced by the rate of evaporation driven by air humidity, as we discussed in the previous section [31,63,68]. These highlight how important it is to consider meteorological and air quality factors like humidity when studying COVID-19 transmission.

### 5.2 Multivariate long short-term memory model

We employed a multivariate Long Short-Term Memory (LSTM) approach to predict COVID-19 cases in Central Thailand. This application of advanced modeling techniques allows for a more comprehensive and accurate forecasting of the dynamics of the pandemic.

Our proposed multi-feature selection technique plays a crucial role in enhancing the efficiency of the LSTM models. By carefully selecting relevant input features and identifying optimal lag times, we provide the LSTM models using only the most pertinent and influential information for accurate predictions. This process effectively reduces the noise or redundancy features. This targeted approach streamlines the model's training process and improves its ability to capture meaningful patterns within the data.

The multivariate LSTM models were effective in capturing the complex relationships of meteorological, particulate matter factors and COVID-19 cases. The selection of LSTM, RNN, and GLM for our study was driven by a pursuit of a comprehensive understanding of diverse modeling approaches in deep learning, machine learning, and statistical approaches. This deliberate choice allowed us to explore the limitations inherent in different methodologies, contributing to a well-rounded evaluation of their effectiveness in predicting COVID-19 dynamics. While all three models demonstrated the capability to predict COVID-19 dynamics, LSTM emerged as the most robust and effective predictive tool. LSTM's superiority lies in its unique architecture, designed to capture intricate temporal dependencies and patterns within the data. The inclusion of memory cells and gating mechanisms enables LSTM to retain and leverage critical information over extended sequences [23,24], proving highly advantageous in the context of the dynamic nature of COVID-19 transmission. Notably, LSTM exhibited consistent and superior performance across both the training and testing datasets. While RNN and GLM contributed valuable insights, LSTM's exceptional performance underscores its significance as the primary model in our study.

### 5.3 Limitation and managerial insights

The main limitation of this study is its exclusive focus on meteorological and particulate matter factors and time lag as determinants influencing COVID-19 dynamics in Central Thailand. While these variables provide valuable insights into virus transmission patterns, it's important to recognize the omission of other crucial factors. These include the closure of facilities due to lockdowns, disruptions in the supply chain, shifts to remote work, and changes in employee productivity due to heightened risk perceptions, which were not addressed in this research. Furthermore, external factors such as human behavior, government policies, and the impact of vaccination efforts may also influence COVID-19 dynamics. The broader socio-economic and operational aspects influenced by the pandemic might play a substantial role in shaping the overall COVID-19 landscape. Additionally, the effectiveness of the LSTM technique used in this study may be limited when applied to datasets with fine-scale or highly fluctuating data, such as hourly data. However, to our knowledge, this research is the first to specifically explore the relationship between meteorological conditions, particulate matter, and COVID-19 cases in Central Thailand. We offer unique insights into how local environmental conditions contribute to the spread and impact of COVID-19.

## 6 Conclusion

Our research on the analysis and prediction of COVID-19 cases in Central Thailand using multivariate long short-term memory (LSTM), incorporating meteorological and particulate matter factors, has yielded significant findings. Our study goes beyond conventional models by incorporating a particular multi-feature selection process. The multi-feature selection process, which involved XGBoost, RF, SBF, PCA, and correlation coefficient analysis, was instrumental in identifying the most crucial input parameters for our predictive model. Among the selected input parameters, relative humidity emerged as a crucial predictor, strengthening our understanding of the virus's transmission dynamics. Our model can demonstrate good performance in predicting COVID-19 cases. This observation is further supported by the high correlation coefficient between relative humidity and the number of COVID-19 cases.

This innovative combination not only enhances the precision of predictions but also provides valuable insights into the intricate relationship between environmental conditions and disease dynamics. Our multivariate LSTM models enabled a comprehensive understanding of the intricate relationships of meteorological, air quality factors, and COVID-19 cases. The LSTM's capacity to handle time-series data and select relevant features proved effective in capturing the complex patterns within the dataset. These innovative methodologies contribute to the advancement of predictive modeling in infectious disease research, offering practical applications for public health interventions and furthering our understanding of the complex factors influencing the spread of COVID-19.

In future research, it is vital to extend the current LSTM-based model by incorporating advanced optimization algorithms [69], such as hybrid heuristics and metaheuristics [70], island algorithms, polyploid algorithms [71], hyper-heuristics [72], and adaptive algorithms [73], to enhance outbreak prediction. A comprehensive discussion should highlight the broader applications of these algorithms in domains like solution approaches, such as online learning, scheduling [69], multi-objective optimization, transportation [70,71], medicine, data classification, and others. Additionally, future studies should explore the endemic perspective of infectious diseases beyond meteorological factors, examining a more comprehensive range of influences on COVID-19 dynamics. Generalizability to other regions, refining predictive models, and investigating long-term effects and recovery phases should be integral to forthcoming research. Collaborations across disciplines can contribute to a more nuanced and interdisciplinary approach to addressing the challenges posed by COVID-19.

## Ethics

The study was conducted in accordance with the Declaration of Helsinki and approved by the Institutional Review Board of Naresuan University (protocol code P1-0078/2566) as Exemption Review.

## Data availability statement

We used data from publicly accessible sources. A summary of their sources and descriptions can be found in the main text and Supplementary File 2.

## CRediT authorship contribution statement

**Chanidapa Winalai:** Writing – review & editing, Writing – original draft, Software, Methodology, Investigation, Formal analysis, Data curation. **Suparinthon Anupong:** Writing – review & editing, Writing – original draft, Investigation, Formal analysis. **Charin Modchang:** Writing – review & editing, Validation, Resources. **Sudarat Chadsuthi:** Writing – review & editing, Writing – original draft, Validation, Supervision, Resources, Project administration, Investigation, Funding acquisition, Conceptualization.

## Declaration of competing interest

The authors declare that they have no known competing financial interests or personal relationships that could have appeared to influence the work reported in this paper.
