# Supplementary material for: LSTM-Powered COVID-19 prediction in central Thailand incorporating meteorological and particulate matter data with a multi-feature selection approach
Source: Heliyon. 2024 Apr 26;10(9):e30319. doi: 10.1016/j.heliyon.2024.e30319 (PMC11070856; doi:10.1016/j.heliyon.2024.e30319)
Supplement: Multimedia component 1 [file mmc1.pdf]

Supplementary file

LSTM-Powered COVID-19 Prediction in Central Thailand Incorporating Meteorological and Particulate Matter Data with a Multi-Feature Selection Approach

Chanidapa Winalai[a], Suparinthon Anupong[b], Charin Modchang[c,d,e], Sudarat Chadsuthi[a*],

[a]Department of Physics, Faculty of Science, Naresuan University, Phitsanulok 65000, Thailand.
[b]Department of Chemistry, Mahidol Wittayanusorn School (MWIT), Salaya, Nakhon Pathom 73170, Thailand
[c]Biophysics Group, Department of Physics, Faculty of Science, Mahidol University, Bangkok 10400, Thailand.
[d]Centre of Excellence in Mathematics, CHE, Bangkok 10400, Thailand
[e]Thailand Center of Excellence in Physics, CHE, 328 Si Ayutthaya Road, Bangkok 10400, Thailand

Supplementary A. Data

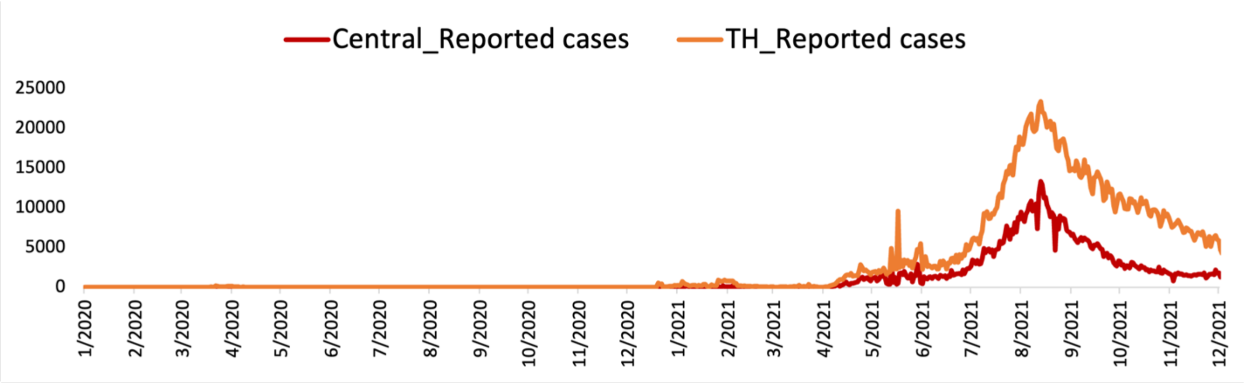

Fig. S1. The number of reported cases in Thailand (orange line) and the central region (red line) from 1st January 2020 to 31st December 2021 (730 days).

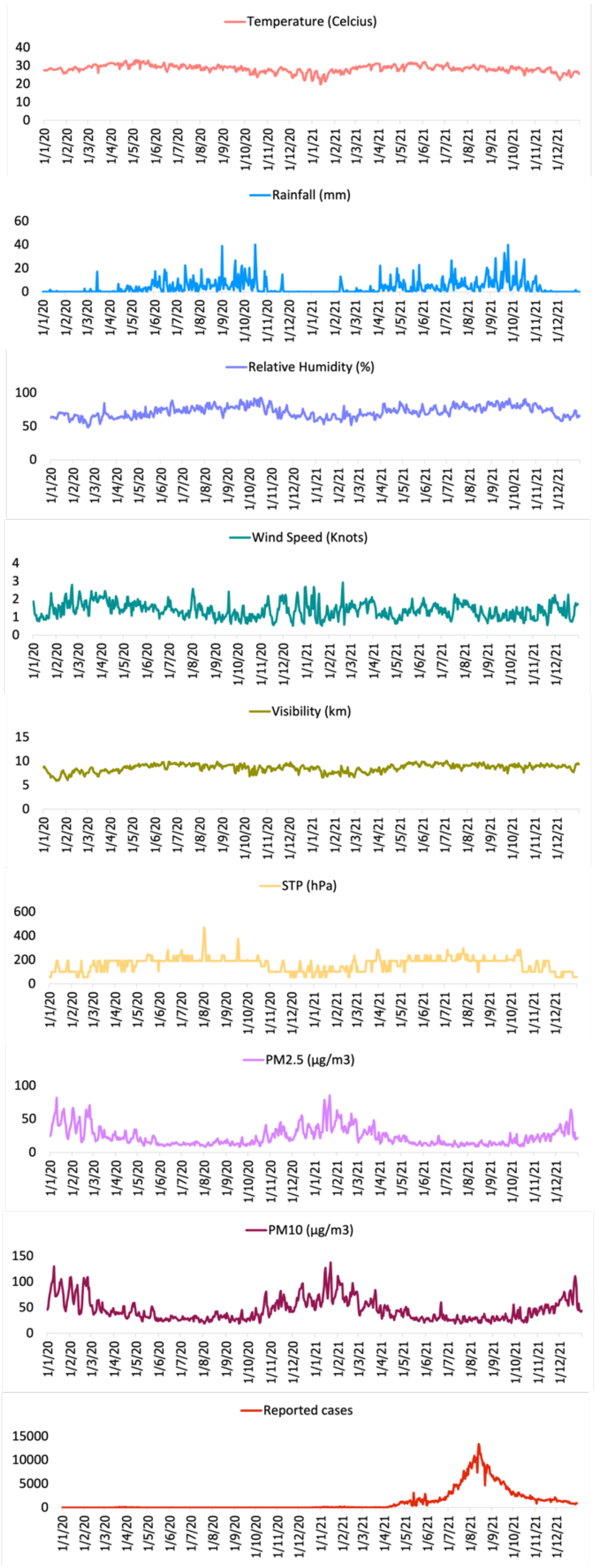

Fig. S2. Time series of meteorological factors, air quality factors, and COVID-19 cases in Central Thailand, spanning from 2020 to December 2021.

Supplementary B. Feature selection results

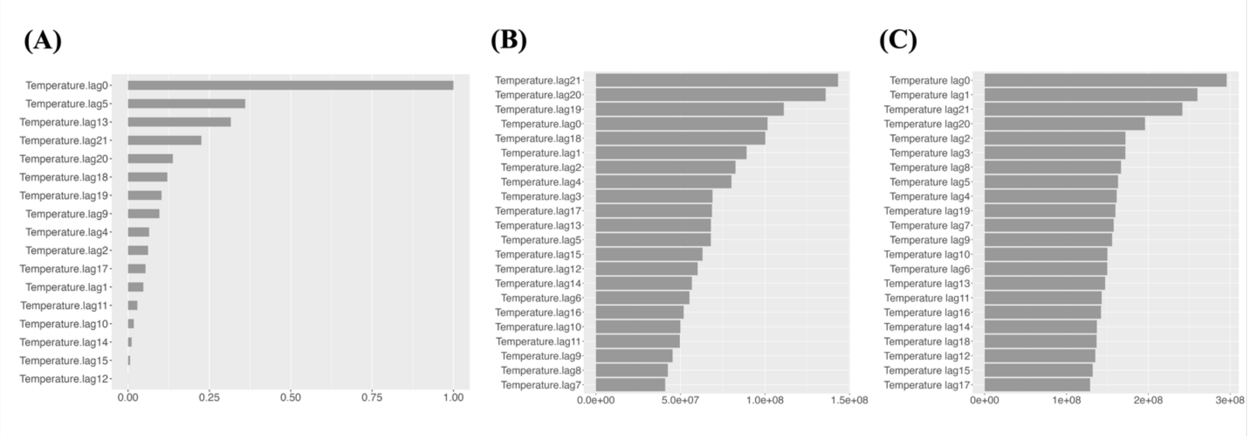

Fig. S3. Lag time assessment of temperature-utilizing (A) XGBoost, (B) Random Forest Model, and (C) SBF techniques, respectively.

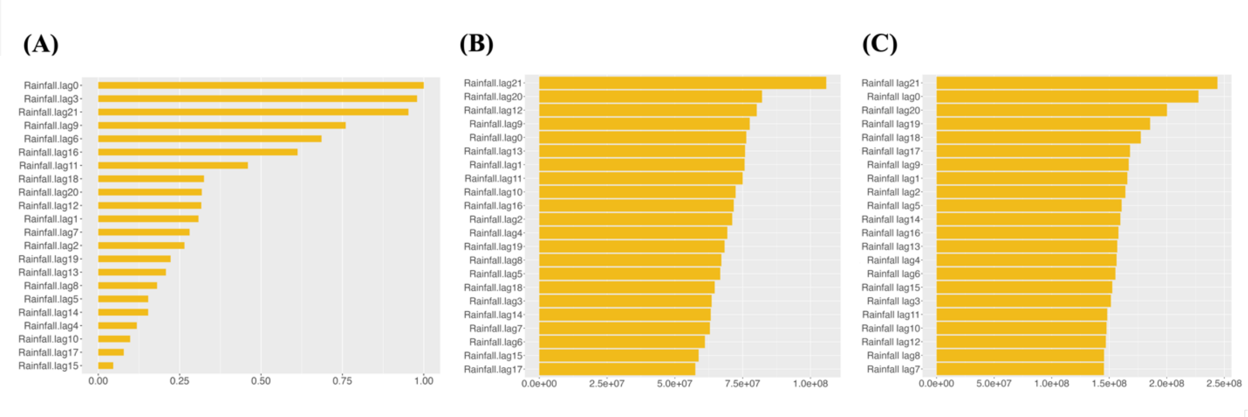

Fig. S4. Lag time assessment of rainfall-utilizing (A) XGBoost, (B) Random Forest Model, and (C) SBF techniques, respectively.

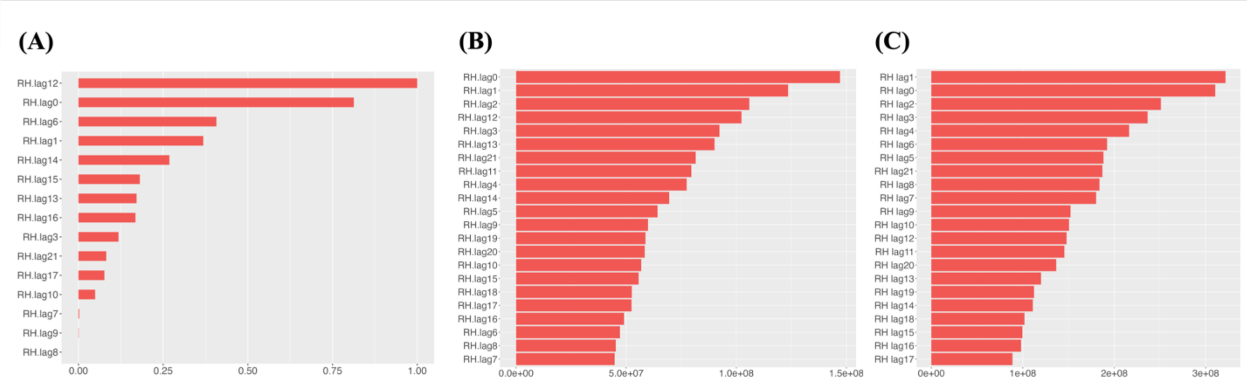

Fig. S5. Lag time assessment of relative humidity (RH)-utilizing (A) XGBoost, (B) Random Forest Model, and (C) SBF techniques, respectively.

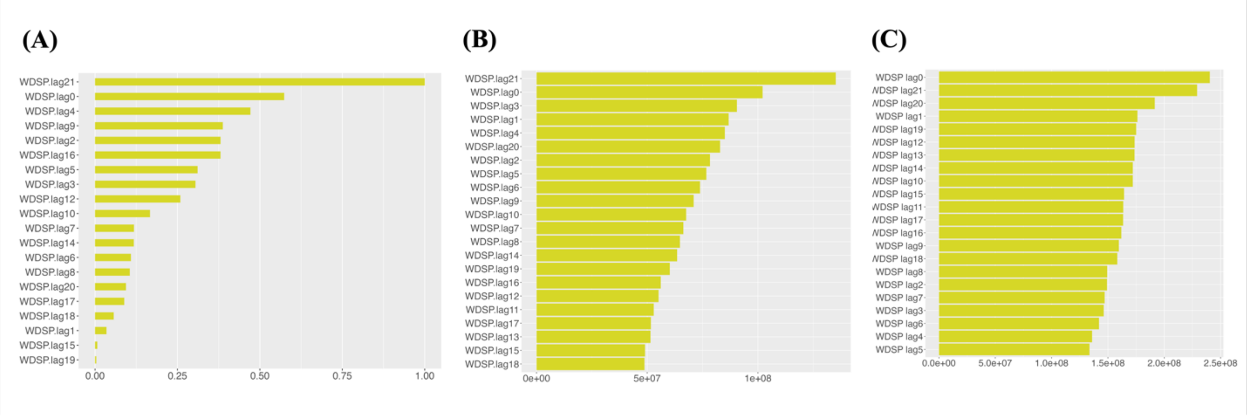

Fig. S6. Lag time assessment of wind speed-utilizing (A) XGBoost, (B) Random Forest Model, and (C) SBF techniques, respectively.

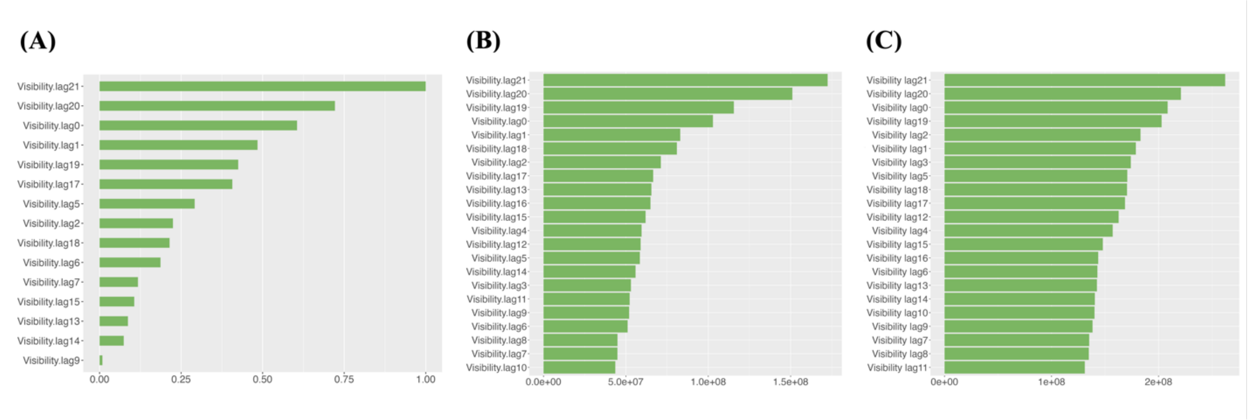

Fig. S7. Lag time assessment of visibility-utilizing (A) XGBoost, (B) Random Forest Model, and (C) SBF techniques, respectively.

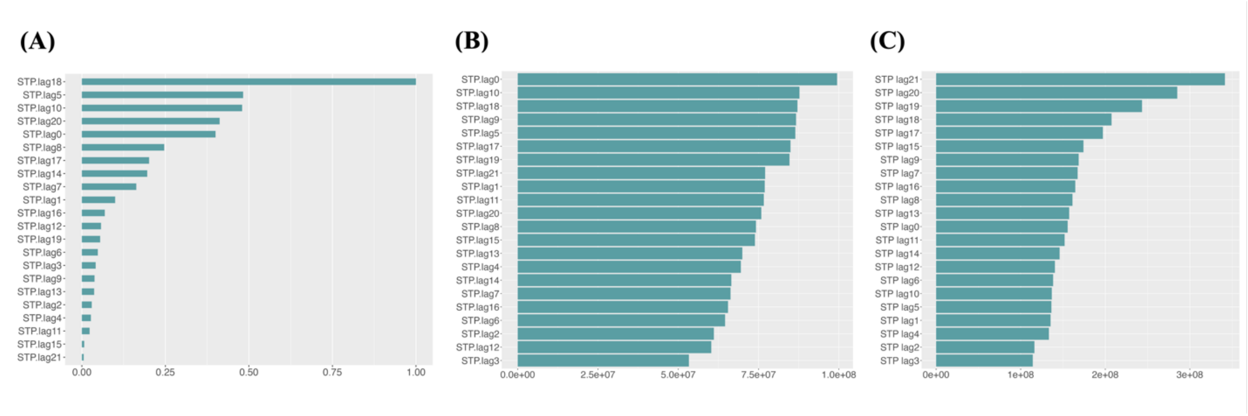

Fig. S8. Lag time assessment of standard pressure (STP)-utilizing (A) XGBoost, (B) Random Forest Model, and (C) SBF techniques, respectively.

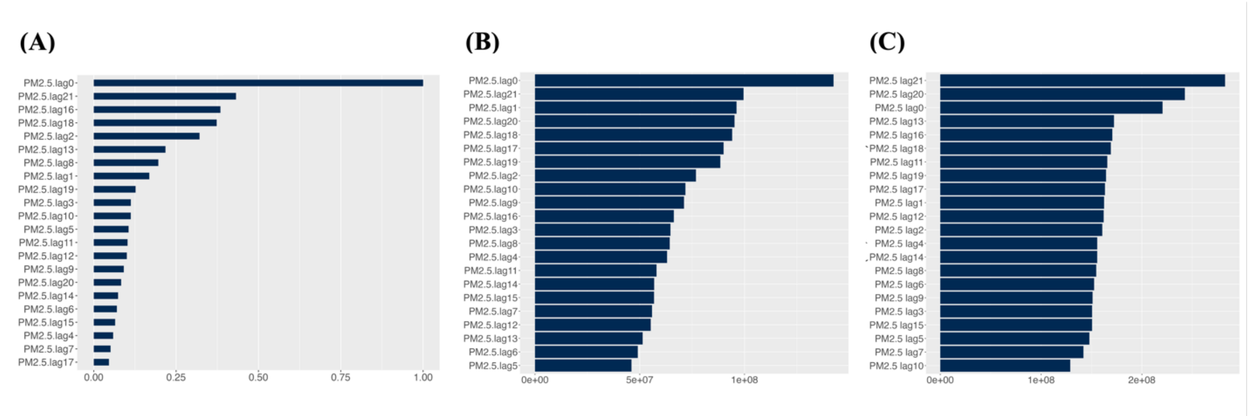

Fig. S9. Lag time assessment of PM2.5-utilizing (A) XGBoost, (B) Random Forest Model, and (C) SBF techniques, respectively.

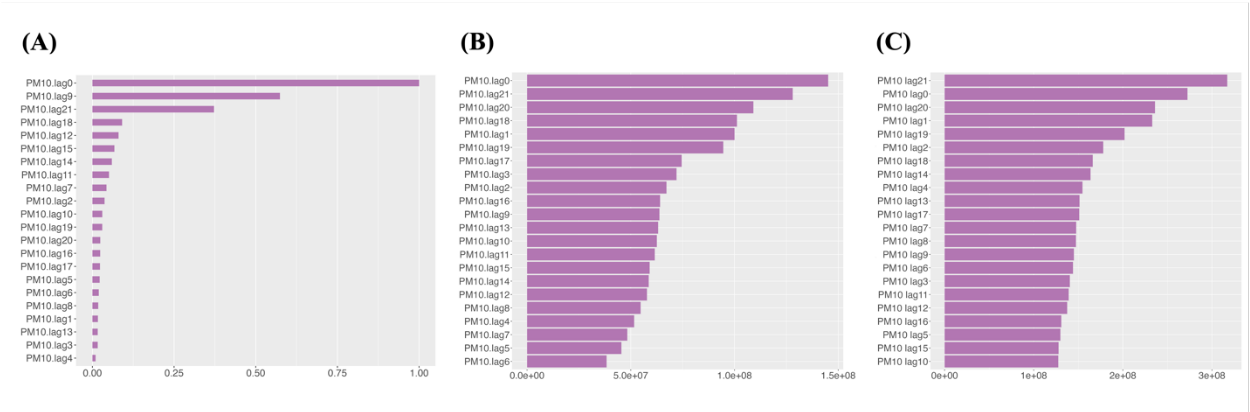

Fig. S10. Lag time assessment of PM10-utilizing (A) XGBoost, (B) Random Forest Model, and (C) SBF techniques, respectively.

Supplementary C. Additional methods and results

Recurrent Neural Network (RNN) Model

A Recurrent Neural Network (RNN) is a type of artificial neural network designed for processing sequential data and capturing patterns in sequences of inputs. Unlike traditional feedforward neural networks, RNNs have connections that form directed cycles, allowing them to maintain a memory of previous inputs in their internal state. This memory enables RNNs to perform tasks that involve sequences and dependencies, making them well-suited for applications in natural language processing, speech recognition, time series analysis, and more [1, 2].

Despite their effectiveness, traditional RNNs have limitations in capturing long-term dependencies. More advanced architectures like LSTMs and Gated Recurrent Units (GRUs) have been developed to overcome these limitations and improve the performance of sequential modeling tasks [1].

Generalized Linear Model (GLM)

A Generalized Linear Model (GLM) is a statistical framework that extends the linear regression model to accommodate a broader range of data distributions and relationships. It is a flexible and versatile approach used for modeling various types of data, including continuous, binary, count, and categorical outcomes. GLMs accommodate different probability distributions for the response variable, extending beyond the normal distribution. Commonly used distributions include the Gaussian (for continuous data), binomial (for binary data), Poisson (for count data), and gamma (for positive continuous data).

GLMs find applications in a wide range of fields, including epidemiology, finance, biology, and social sciences. They are particularly useful when dealing with non-normally distributed or categorical response variables [3].

We assess the efficiency of the LSTM model by comparing it with both the Recurrent Neural Network (RNN) and the Generalized Linear Model (GLM). The comparison of diverse machine learning and statistical techniques contributes to evaluating their effectiveness in predicting or analyzing the target variables. The results, including Mean Absolute Error (MAE) and Root Mean Square Error (RMSE) for the three models, are presented in Tables S1 and S2, focusing on the training and the testing datasets, respectively.

References

1. Dudek, G., S. Smyl, and P. Pełka. *Recurrent Neural Networks for Forecasting Time Series with Multiple Seasonality: A Comparative Study*. in *Theory and Applications of Time Series Analysis*. 2023. Cham: Springer Nature Switzerland.
2. Wang, J., et al., *NGCU: A New RNN Model for Time-Series Data Prediction.* Big Data Research, 2022. **27**: p. 100296.

3. Beard, E., et al., *Understanding and using time series analyses in addiction research.* Addiction, 2019. **114**(10): p. 1866-1884.

Table S1. Mean Absolute Error (MAE) and Root Mean Square Error (RMSE) of the training dataset

**Model: Input features**	**LSTM**		**RNN**		**GLM**	
	**MAE**	**RMSE**	**MAE**	**RMSE**	**MAE**	**RMSE**
A) Cases (1-day lag)	21.18	58.61	35.48	96.65	38.55	106.71
B) Relative Humidity (0-day lag), Cases (1-day lag)	19.49	53.30	28.99	76.13	33.92	94.95
C) Wind speed (0-day lag), Cases (1-day lag)	20.00	53.80	33.90	94.67	37.03	104.20
D) Visibility (0-day lag), Cases (1-day lag)	23.16	67.31	39.73	110.79	41.56	119.44
E) PM10 (21-day lag), Cases (1-day lag)	21.81	60.42	38.90	107.05	40.77	115.45
F) Relative Humidity (0-day lag), Wind speed (0-day lag), Visibility (0-day lag), PM10 (21-day lag), Cases (1-day lag)	25.05	73.37	39.98	109.81	42.96	120.07

102 **Table S2.** Mean Absolute Error (MAE) and Root Mean Square Error (RMSE) of the testing dataset

**Model: Input features**	**LSTM**		**RNN**		**GLM**	
	**MAE**	**RMSE**	**MAE**	**RMSE**	**MAE**	**RMSE**
A) Cases (1-day lag)	65.90	100.21	78.16	144.16	85.98	154.30
B) Relative Humidity (0-day lag), Cases (1-day lag)	58.14	79.87	75.51	129.79	80.93	147.69
C) Wind speed (0-day lag), Cases (1-day lag)	63.55	96.77	77.18	139.35	83.78	152.27
D) Visibility (0-day lag), Cases (1-day lag)	70.53	109.71	85.98	156.76	87.86	165.20
E) PM10 (21-day lag), Cases (1-day lag)	66.10	98.60	89.63	163.02	90.40	171.83
F) Relative Humidity (0-day lag), Wind speed (0-day lag), Visibility (0-day lag), PM10 (21-day lag), Cases (1-day lag)	73.93	123.26	91.51	156.48	94.65	172.84

103

104

105

106

Long Short-Term Memory (LSTM) Model Performance

Based on the selected input parameters from the multi-feature selection stage, we constructed multivariate LSTM models. These models were designed to capture the complex relationships between meteorological factors and COVID-19 cases, enabling us to make accurate predictions over time.

Fig. S11 displays the performance of the models A – F. Among the selected input parameters, relative humidity emerged as the most influential factor in predicting COVID-19 cases. This finding provides compelling evidence that relative humidity significantly impacts the transmission dynamics of the virus in Central Thailand.

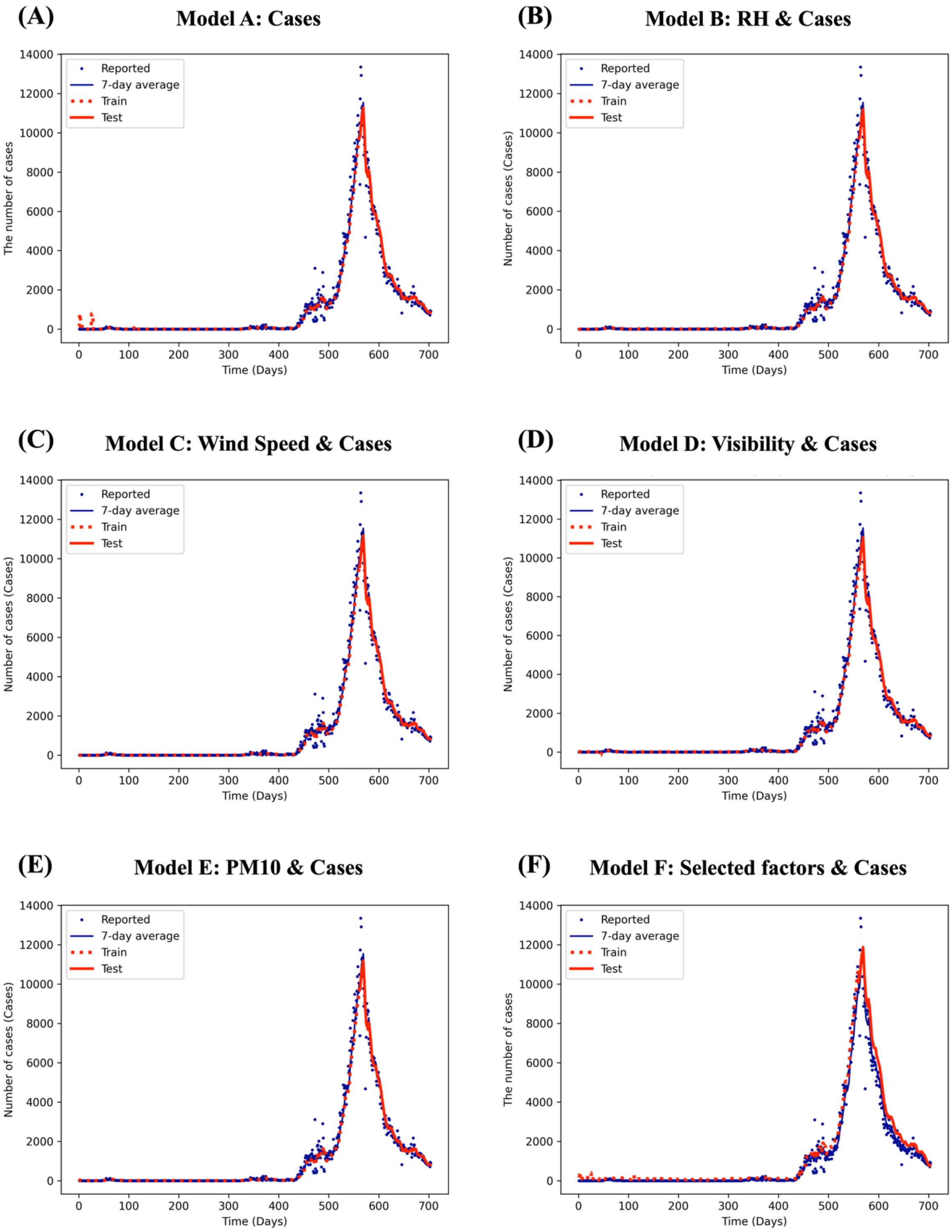

Fig. S11. (A) – (F) COVID-19 case predictions over time (model A – F, respectively), with illustrations of reported COVID-19 cases (represented as blue dots), the 7-day moving average of cases (represented as a blue line), training results (shown as red dashed lines), and testing data (depicted as red lines).

Supplementary D. Code complements

In this section, we provide supplementary code detailing the implementation of key processes and techniques utilized in our research on analyzing and predicting COVID-19 dynamics in Central Thailand. This code complements the main research methodology and results, offering a more in-depth view of our technical approach.

XGBoost in R

```
library(xgboost)
xgb_train = xgb.DMatrix(data = x_train, label = COVID_train)
xgb_test = xgb.DMatrix(data = x_val, label = COVID_val)

watchlist = list(train = xgb_train, test = xgb_test)
model = xgb.train(data = xgb_train, max.depth = 3, watchlist = watchlist, nrounds = 100)
model_xgboost = xgboost(data = xgb_train, max.depth = 3, nrounds = #least error round, verbose = 0)
summary(model_xgboost)

importance_matrix = xgb.importance(colnames(xgb_train), model = model_xgboost)
imp_score = xgb.ggplot.importance(importance_matrix, rel_to_first = TRUE,n_clusters = c(1:3))
```

Random Forest in R

```
library(randomForest)
rf = randomForest(x = train, y = COVID_train, xtest = val, ytest = COVID_val, ntree = 500)
var_imp <- varImp(rf, scale = FALSE)
var_imp_df <- data.frame(cbind(variable = rownames(var_imp), score = var_imp[,1]))
var_imp_df$score <- as.double(var_imp_df$score)
var_imp_df[order(var_imp_df$score,decreasing = TRUE),]
```

SBF in R

```
library(gam)
library(caret)
filterCtrl <- sbfControl(functions = rfSBF, method = "repeatedcv", repeats = 100)
SBF <- sbf(x = data, y= COVID, sbfControl = filterCtrl)
SBF_score = as.numeric(SBF$fit$importance)
```

```
Filter_sbf = cbind(SBF$optVariables, SBF_score)
Filter_sbf = as.data.frame(rfWithFiltersbf)
Filter_sbf$SBF_score = as.numeric(Filter_sbf$SBF_score)
```

PCA in R

```
library(FactoMineR)
library("factoextra")
PCA_data = PCA(data)
PCA_plt = fviz_pca_var(PCA_data)
```

Correlation in R

```
library(corrplot)
cor.data_select = cor(data, use = "pairwise", method = "spearman")
corr = corrplot(cor.data, method = "number", type = "upper", title = "")
```

LSTM model in Python

```
import numpy as np
import pandas as pd
from sklearn.model_selection import train_test_split
from tensorflow import keras
from tensorflow.keras.models import Sequential
from tensorflow.keras.layers import LSTM, Dense
from sklearn.metrics import mean_absolute_error

# Load the dataset
data = pd.read_csv('covid_data.csv')

# Split data into training and testing sets
X_train, X_test, y_train, y_test = train_test_split(features, target, test_size=0.2, random_state=42)

# Build the LSTM model
```

```
def MODEL_LSTM(name,x_train,x_test,y_train,y_test,Num_Exp,n_steps_in,n_steps_out,Epochs,Hidden,pred_days,pred_batch,Batch_size):

    model = Sequential()
    model.add(LSTM(Hidden, activation='relu', input_shape=(n_steps_in,n_features), dropout=0.2))
    model.add(Dense(32))
    model.add(Dense(n_steps_out))
    model.compile(optimizer='adam', loss='mae')

    # fit model
    model.fit(x_train, y_train, epochs=Epochs,batch_size=Batch_size, verbose=0, shuffle=False)
    model.summary()
    model.save('covid_lstm_model.h5')
    model = keras.models.load_model('D:/COVID_Multivariate/Data/model/' + name + '(21,1)_lstm.h5')
    Best_MAE=10000000000   #Assigning a large number
    Best_Train_MAE = 10000000000
    start_time=time.time()
    final_predictions=np.zeros([1,Num_Exp,pred_days])
    final_step_01 = np.zeros([Num_Exp,pred_days])

    for run in range(Num_Exp):
        print("Experiment",run+1,"in progress")
        # fit model
        model.fit(x_train, y_train, epochs=Epochs,batch_size=Batch_size, verbose=0, shuffle=False)
        y_predicttrain = model.predict(x_train)/10000
        y_predicttest = model.predict(x_test)/10000
        print(y_predicttest.shape)
        train_acc[run] = mae( y_predicttrain,y_train)
        test_acc[run] = mae( y_predicttest, y_test)

        if train_acc[run] < Best_Train_MAE:
            Best_Train_MAE = train_acc[run]
            Best_Predict_Train = y_predicttrain
```

```
        if test_acc[run]<Best_MAE:
            Best_MAE=test_acc[run]
            Best_Predict_Test=y_predicttest
        for j in range(n_steps_out):
            Step_MAE[run][j]=mae(y_predicttest[:,j], y_test[:,j])

        From_Predict = copy.deepcopy(y_predicttest)
        From_Test = copy.deepcopy(x_test)
        step_01 = np.array([])

        predictions = np.zeros([70,n_steps_out])

    print("Total time for",Num_Exp,"experiments",time.time()-start_time)
    return train_acc,test_acc,Step_MAE,Best_Predict_Test,Best_Predict_Train,final_predictions

# Calculate Mean Absolute Error
def mae(pred, actual):
    pred_flat1 = np.ndarray.flatten(pred)
    pred_un1 = scaler.inverse_transform(np.reshape(pred_flat1,(pred_flat1.shape[0],1)))
    actual_flat1 = np.ndarray.flatten(actual)
    actual_un1 = scaler.inverse_transform(np.reshape(actual_flat1,(actual_flat1.shape[0],1)))
    actual_flaten1 = []
    pred_flaten1 = []
    for lis in actual_un1:
      actual_flaten1.append(lis[0])
    for lis in pred_un1:
      pred_flaten1.append(lis[0])
    error = np.subtract(pred_flaten1, actual_flaten1)
    # print(error)
    try:
      error = np.reshape(error,(actual.shape[0],actual.shape[1]))
    except:
      pass
```

```
    sqerror1= np.sum(abs(error))/actual.shape[0]
    return np.sqrt(sqerror1)
mae = mean_absolute_error(y_test, y_pred)
print(f"Mean Absolute Error: ")
```
