# Supplementary material for: LSTM-Powered COVID-19 prediction in central Thailand incorporating meteorological and particulate matter data with a multi-feature selection approach
Source: Heliyon. 2024 Apr 26;10(9):e30319. doi: 10.1016/j.heliyon.2024.e30319 (PMC11070856; doi:10.1016/j.heliyon.2024.e30319)
Supplement: Multimedia component 2 [file mmc2.pdf]

Supplementary file 2

LSTM-Powered COVID-19 Prediction in Central Thailand Incorporating Meteorological and Particulate Matter Data with a Multi-Feature Selection Approach

Chanidapa Winalai[a], Suparinthon Anupong[b], Charin Modchang[c,d,e], Sudarat Chadsuthi[a*],

[a]Department of Physics, Faculty of Science, Naresuan University, Phitsanulok 65000, Thailand.

[b]Department of Chemistry, Mahidol Wittayanusorn School (MWIT), Salaya, Nakhon Pathom 73170, Thailand

[c]Biophysics Group, Department of Physics, Faculty of Science, Mahidol University, Bangkok 10400, Thailand.

[d]Centre of Excellence in Mathematics, CHE, Bangkok 10400, Thailand

[e]Thailand Center of Excellence in Physics, CHE, 328 Si Ayutthaya Road, Bangkok 10400, Thailand

DATE	RH	WDSP	VISIB	TEMP	STP	PRCP	PM25	PM10	CASES CENTRAL	CASES TH
01/01/2020	62.85	1.88	8.69	28.08	13.62	0.00	76.94	45.75	0	0
02/01/2020	64.08	1.61	8.92	27.78	13.76	0.00	81.24	50.18	0	0
03/01/2020	63.42	1.19	8.60	27.89	11.76	0.00	93.94	63.90	0	0
04/01/2020	64.21	1.08	8.48	27.97	9.91	0.00	109.41	73.68	0	0
05/01/2020	63.53	1.12	8.26	27.91	10.14	0.00	115.76	79.76	0	0
06/01/2020	62.14	0.82	7.95	28.15	9.93	0.00	124.75	91.10	0	0
07/01/2020	62.98	0.79	7.63	28.40	9.31	0.00	135.44	98.03	0	0
08/01/2020	62.79	0.91	7.43	28.51	8.72	0.00	138.19	99.48	0	0
09/01/2020	60.70	0.76	7.46	29.06	8.12	0.00	136.06	105.93	0	0
10/01/2020	63.83	0.99	7.22	29.22	63.11	0.00	154.63	130.14	0	0
11/01/2020	65.84	0.97	6.56	29.08	112.47	2.06	132.88	89.10	0	0
12/01/2020	69.91	1.19	6.92	28.77	111.94	0.00	118.94	71.86	1	1
13/01/2020	70.02	1.04	6.92	28.62	60.19	0.98	116.06	71.59	0	0
14/01/2020	70.93	1.06	6.81	28.34	61.04	0.00	117.19	72.90	0	0
15/01/2020	69.49	0.85	6.64	28.25	8.74	0.00	116.69	76.17	0	0
16/01/2020	69.06	0.90	6.50	28.31	9.40	0.00	124.19	79.76	0	0
17/01/2020	70.30	0.90	6.29	28.42	9.81	0.00	130.75	87.07	1	1
18/01/2020	69.62	0.85	6.04	28.59	9.06	0.00	147.00	99.00	0	0
19/01/2020	69.27	1.10	5.94	28.86	9.68	0.00	146.00	96.55	0	0
20/01/2020	66.60	1.01	6.16	28.94	10.97	0.89	149.69	105.21	0	0
21/01/2020	69.43	0.93	6.04	28.73	11.77	0.00	141.06	91.76	0	0
22/01/2020	69.60	0.94	6.00	29.03	10.59	0.00	123.56	83.52	1	2
23/01/2020	69.66	1.51	6.47	29.29	9.18	0.00	117.00	74.03	0	0
24/01/2020	69.45	1.71	6.62	29.21	9.00	0.00	89.69	52.48	0	1
25/01/2020	68.79	2.34	7.17	28.56	60.25	0.00	73.65	46.14	0	1
26/01/2020	60.73	1.83	7.90	27.92	111.99	0.00	67.41	42.45	0	2
27/01/2020	56.06	1.67	7.67	27.44	111.86	0.00	80.94	51.55	0	0
28/01/2020	58.34	1.33	8.15	26.49	8.28	0.00	98.88	62.21	0	6
29/01/2020	61.08	1.16	7.54	27.09	9.61	0.00	103.24	69.97	0	0
30/01/2020	61.32	1.51	7.44	27.13	9.60	0.00	110.82	79.69	0	0
31/01/2020	59.33	1.48	6.98	26.53	10.52	0.00	122.89	88.21	1	5
01/02/2020	61.47	1.07	6.66	26.97	10.52	0.00	148.11	107.17	0	0
02/02/2020	64.45	0.90	6.35	27.72	11.36	0.00	150.56	109.28	0	0
03/02/2020	67.68	1.28	6.05	28.10	10.00	0.00	143.72	98.69	0	0
04/02/2020	65.78	1.68	6.65	28.65	9.26	0.00	126.50	80.69	3	6
05/02/2020	66.55	1.44	7.10	28.15	9.02	0.00	102.89	64.55	0	0
06/02/2020	66.62	1.60	7.22	28.41	9.82	0.04	89.44	56.76	0	0
07/02/2020	66.20	1.00	6.79	28.76	10.26	0.15	100.67	65.69	0	0
08/02/2020	65.99	1.14	6.81	28.55	11.18	0.00	118.61	78.00	2	7
09/02/2020	58.40	1.79	7.59	28.26	11.46	0.00	119.67	83.86	0	0

10/02/2020	55.68	1.62	8.02	28.54	11.43	0.00	113.06	86.31	0	0
11/02/2020	56.83	1.13	7.63	29.74	10.79	0.00	136.17	93.93	0	1
12/02/2020	65.65	2.01	7.75	29.71	9.98	0.00	115.18	73.41	0	0
13/02/2020	65.31	2.21	8.33	29.66	8.45	0.00	59.00	37.79	0	0
14/02/2020	64.83	2.31	8.25	29.15	112.37	0.00	57.06	36.72	0	0
15/02/2020	54.47	1.81	8.52	28.44	8.25	0.00	72.33	43.83	1	1
16/02/2020	57.16	1.51	8.23	28.36	9.70	0.00	64.83	38.90	0	0
17/02/2020	60.00	1.62	8.17	29.30	10.74	0.00	76.67	50.31	0	1
18/02/2020	57.79	1.93	7.91	27.29	12.21	0.00	103.44	75.69	0	0
19/02/2020	55.27	1.51	7.69	28.10	12.03	0.00	133.50	96.38	0	0
20/02/2020	52.28	1.76	7.38	28.77	11.79	0.00	144.00	107.52	0	0
21/02/2020	48.73	2.57	7.96	28.59	14.09	0.00	134.89	96.14	0	0
22/02/2020	48.30	2.80	8.22	28.56	13.70	0.00	130.61	97.48	0	0
23/02/2020	52.11	1.81	7.88	28.24	12.53	0.00	128.11	91.93	0	0
24/02/2020	51.47	1.28	7.75	28.37	9.97	0.00	146.33	104.97	0	0
25/02/2020	57.89	1.35	7.20	28.67	9.13	0.00	151.22	109.03	2	2
26/02/2020	62.41	1.17	6.81	28.97	9.02	0.00	130.06	85.38	3	3
27/02/2020	64.32	1.20	7.11	28.97	61.11	3.20	126.89	88.90	0	0
28/02/2020	66.04	1.53	7.47	29.22	60.41	0.13	100.78	60.14	1	1
29/02/2020	66.05	1.46	7.63	29.63	63.52	0.09	89.17	52.45	1	1
01/03/2020	64.63	1.69	7.65	30.17	63.47	0.00	85.44	53.55	0	0
02/03/2020	64.99	2.15	7.57	30.21	112.54	0.00	88.89	54.17	1	1
03/03/2020	65.81	2.44	7.69	30.27	112.41	0.00	74.67	45.64	0	0
04/03/2020	66.06	2.30	8.25	30.30	60.21	0.00	60.89	36.25	0	0
05/03/2020	68.46	1.96	8.48	30.32	60.76	0.00	65.00	39.97	0	4
06/03/2020	69.42	1.77	8.73	29.97	9.46	1.31	63.00	38.72	0	1
07/03/2020	68.34	1.59	8.63	30.06	8.81	1.16	70.83	40.69	2	2
08/03/2020	59.64	1.68	8.61	30.67	111.43	0.00	81.06	46.03	0	0
09/03/2020	53.54	1.40	7.95	31.01	109.80	0.00	112.28	63.69	0	0
10/03/2020	58.86	1.90	7.70	30.89	110.55	0.00	106.22	61.17	3	3
11/03/2020	58.08	1.83	7.60	31.24	112.21	0.00	109.67	64.71	5	6
12/03/2020	64.24	1.94	7.44	31.14	8.65	0.00	96.67	53.10	13	13
13/03/2020	65.75	1.53	7.46	30.70	7.73	0.04	81.78	45.97	3	3
14/03/2020	71.23	1.58	7.39	30.18	63.26	0.09	75.22	42.83	6	7
15/03/2020	84.54	1.06	6.84	25.87	9.47	19.67	77.78	40.15	29	32
16/03/2020	72.04	1.08	6.83	29.21	9.71	2.03	99.28	60.07	23	33
17/03/2020	69.50	1.86	7.37	30.11	9.16	0.00	76.72	46.83	20	30
18/03/2020	67.93	2.07	7.54	30.38	112.44	0.00	76.39	46.55	26	35
19/03/2020	68.69	2.46	8.04	30.31	112.37	0.00	75.39	45.00	44	60
20/03/2020	67.77	2.17	7.94	30.31	63.85	0.10	73.94	42.28	36	50
21/03/2020	65.61	1.96	8.11	30.35	8.36	0.00	76.00	43.03	51	89

22/03/2020	62.50	1.91	8.03	30.60	63.42	0.00	70.44	38.17	117	188
23/03/2020	61.80	2.11	8.06	30.74	112.05	0.00	70.83	40.14	77	122
24/03/2020	62.75	2.07	7.99	30.62	110.89	0.00	68.94	36.90	52	106
25/03/2020	65.32	2.37	8.37	30.48	111.19	0.00	69.50	36.76	67	107
26/03/2020	63.07	1.92	8.33	30.82	112.24	0.00	65.61	32.96	64	111
27/03/2020	61.46	1.82	8.12	31.29	111.92	0.00	69.44	34.00	53	91
28/03/2020	60.04	1.87	7.98	31.24	110.73	0.00	84.17	43.48	67	108
29/03/2020	60.96	2.04	7.63	31.14	110.43	0.00	77.22	40.50	113	141
30/03/2020	60.88	2.20	7.68	31.33	111.15	0.00	85.00	44.37	98	138
31/03/2020	60.98	2.07	7.51	31.58	111.00	0.00	82.00	48.57	91	128
01/04/2020	62.06	2.14	7.80	31.71	111.07	0.00	78.72	41.90	81	120
02/04/2020	61.60	2.10	7.90	31.70	111.70	0.00	71.67	38.31	41	104
03/04/2020	63.63	2.13	7.97	31.57	112.66	0.00	65.94	36.04	57	103
04/04/2020	62.10	2.03	7.83	31.84	112.57	0.05	69.22	41.29	51	89
05/04/2020	63.69	2.10	8.27	31.74	60.49	0.03	66.61	37.93	41	102
06/04/2020	63.50	2.45	8.16	31.65	111.94	0.62	65.00	37.66	34	51
07/04/2020	64.93	2.37	8.05	31.43	111.24	0.00	70.89	40.28	21	38
08/04/2020	64.85	2.13	8.17	31.27	112.33	0.47	66.72	37.50	32	111
09/04/2020	63.85	1.83	8.31	31.37	60.63	0.01	67.06	38.97	27	54
10/04/2020	64.47	1.73	8.34	31.03	9.06	0.14	72.83	38.86	26	50
11/04/2020	62.22	1.60	8.05	31.74	60.81	0.36	74.89	39.90	16	45
12/04/2020	62.37	2.09	8.37	31.71	112.19	0.08	69.22	37.41	15	33
13/04/2020	73.05	1.65	7.45	28.63	61.16	7.82	80.39	41.54	11	28
14/04/2020	68.35	1.22	7.67	29.73	9.18	1.41	97.72	52.43	10	34
15/04/2020	63.13	1.20	7.82	31.53	60.41	0.00	95.44	52.48	15	30
16/04/2020	65.29	1.94	8.19	31.06	112.19	1.12	68.56	35.52	17	29
17/04/2020	67.00	1.95	8.10	30.27	60.61	2.57	61.17	31.29	17	28
18/04/2020	64.24	1.47	8.50	31.19	60.64	0.05	56.00	29.11	25	33
19/04/2020	62.93	1.68	8.69	31.62	112.51	0.24	55.00	28.41	23	32
20/04/2020	60.77	1.60	8.83	32.11	112.21	0.00	61.06	31.93	13	27
21/04/2020	59.50	2.17	8.97	32.82	111.24	0.00	58.11	30.81	11	19
22/04/2020	59.03	2.03	8.67	33.10	111.14	0.00	71.44	38.39	6	15
23/04/2020	62.73	1.85	8.59	31.36	111.98	2.53	71.28	40.82	6	13
24/04/2020	63.87	1.53	8.99	30.92	112.51	1.40	72.72	40.29	4	15
25/04/2020	73.65	1.51	8.67	28.67	8.51	6.03	75.33	43.46	2	53
26/04/2020	63.97	1.20	8.69	30.98	60.68	5.04	87.22	51.93	8	15
27/04/2020	72.36	1.40	8.45	28.81	10.17	3.61	96.06	58.39	3	9
28/04/2020	60.32	1.39	9.11	31.17	60.37	0.56	84.39	52.41	2	7
29/04/2020	66.67	1.41	8.37	30.74	60.12	4.75	100.28	59.04	5	9
30/04/2020	66.96	1.27	8.33	31.14	60.00	3.56	82.50	47.21	3	7
01/05/2020	69.63	1.44	8.68	30.72	111.66	3.60	70.44	36.71	1	6

02/05/2020	66.05	1.35	8.56	31.59	110.60	1.17	64.28	34.75	3	6
03/05/2020	63.60	1.74	8.95	32.68	110.47	0.00	60.94	33.86	2	3
04/05/2020	70.72	1.48	8.62	31.11	111.58	1.10	67.50	38.00	0	18
05/05/2020	61.83	1.54	9.08	33.14	109.95	0.11	76.17	40.74	0	1
06/05/2020	62.79	2.13	9.13	33.22	109.51	0.00	66.33	34.30	1	1
07/05/2020	67.20	2.14	9.00	31.97	109.52	5.42	57.67	31.81	2	3
08/05/2020	68.78	1.63	9.10	31.83	109.65	1.10	59.67	31.88	0	8
09/05/2020	64.97	1.73	9.17	32.96	110.24	0.00	47.22	24.44	1	4
10/05/2020	64.65	1.77	9.49	32.94	110.38	0.00	43.28	22.96	2	5
11/05/2020	79.43	1.53	8.83	28.12	112.34	4.98	52.72	30.89	0	6
12/05/2020	64.47	1.08	8.97	31.31	111.42	1.77	71.00	43.64	1	2
13/05/2020	63.96	1.82	9.19	32.35	110.60	0.03	61.83	33.57	0	0
14/05/2020	67.08	1.37	9.21	31.73	110.37	2.31	60.39	32.64	0	1
15/05/2020	64.15	1.74	9.28	32.42	160.94	0.17	59.06	31.14	0	7
16/05/2020	62.92	1.90	9.40	32.86	160.38	0.14	58.67	31.79	0	0
17/05/2020	69.83	1.77	9.13	31.71	169.30	3.31	59.17	31.07	0	3
18/05/2020	72.42	1.60	9.20	31.28	160.31	3.06	59.11	31.43	2	3
19/05/2020	69.80	1.65	9.33	31.54	161.02	2.21	50.39	29.21	0	2
20/05/2020	68.71	2.00	9.48	31.70	161.57	2.02	55.28	30.63	1	1
21/05/2020	67.23	1.69	9.14	32.22	109.37	0.33	63.17	34.75	1	3
22/05/2020	63.38	1.67	9.22	33.13	161.47	0.01	79.11	47.41	0	0
23/05/2020	68.61	1.89	8.97	32.14	109.38	2.86	80.83	52.19	0	3
24/05/2020	75.94	1.60	8.72	30.77	111.02	4.11	77.50	48.56	0	0
25/05/2020	78.67	1.41	8.71	29.92	112.56	1.70	73.44	44.07	0	2
26/05/2020	77.82	1.31	8.85	29.70	112.48	3.90	71.12	41.67	2	3
27/05/2020	69.25	1.65	9.46	31.22	111.47	1.85	52.89	28.00	1	9
28/05/2020	73.01	1.53	9.27	30.54	111.46	4.22	49.83	25.18	11	11
29/05/2020	75.37	1.56	8.90	29.90	111.85	10.02	57.33	29.96	11	11
30/05/2020	76.71	1.85	9.27	29.67	112.88	8.17	48.44	24.46	0	1
31/05/2020	71.77	1.30	9.69	30.66	8.16	7.87	49.44	23.52	1	4
01/06/2020	70.31	1.54	9.47	31.31	112.13	4.95	53.39	28.57	0	1
02/06/2020	78.31	1.52	8.98	29.09	111.62	20.02	49.28	25.00	0	1
03/06/2020	77.00	1.47	9.36	29.10	110.39	8.76	49.00	24.96	0	1
04/06/2020	74.55	1.42	9.44	30.01	109.84	5.35	50.50	28.96	15	17
05/06/2020	76.55	1.53	9.36	29.71	109.94	3.04	47.83	27.63	1	1
06/06/2020	75.71	1.61	9.02	29.60	109.96	9.86	45.17	23.85	1	2
07/06/2020	76.37	1.60	9.23	29.66	109.47	5.58	40.83	23.00	8	8
08/06/2020	76.41	1.71	9.14	29.15	109.27	15.28	43.89	24.79	7	7
09/06/2020	77.90	1.70	9.22	29.16	109.62	3.78	45.39	26.32	1	2
10/06/2020	74.62	1.30	9.46	29.63	110.23	0.96	45.94	25.25	2	4
11/06/2020	70.45	1.59	9.79	30.55	115.26	0.14	52.50	29.32	0	0

12/06/2020	66.87	1.69	9.64	31.37	169.01	0.10	48.28	26.21	0	4
13/06/2020	65.92	1.60	9.83	32.10	212.01	0.00	47.28	28.00	5	5
14/06/2020	74.15	1.49	8.93	30.42	108.37	12.80	59.06	28.04	1	1
15/06/2020	85.41	1.38	8.34	27.97	110.59	20.41	45.28	23.39	0	0
16/06/2020	84.39	1.42	8.49	27.90	111.13	10.63	49.28	24.36	0	0
17/06/2020	88.75	1.37	8.25	26.85	111.40	17.78	55.28	26.36	0	0
18/06/2020	81.57	0.91	8.60	28.65	110.96	4.52	59.11	34.18	5	6
19/06/2020	78.97	1.19	9.10	29.13	110.38	3.46	55.44	32.50	5	5
20/06/2020	69.87	1.19	9.91	31.01	109.51	0.03	53.78	32.63	0	1
21/06/2020	71.27	1.26	9.86	31.41	160.75	2.97	53.33	28.56	0	1
22/06/2020	74.22	1.20	9.34	30.38	108.83	7.11	59.28	31.36	0	3
23/06/2020	75.07	1.17	9.51	30.38	110.24	0.37	57.50	31.07	0	5
24/06/2020	71.59	1.33	9.53	31.26	109.59	2.20	62.50	31.57	1	1
25/06/2020	74.31	1.37	9.45	30.73	161.65	2.33	53.67	29.32	0	1
26/06/2020	75.42	1.58	9.53	29.76	109.56	5.91	50.89	28.25	2	4
27/06/2020	73.02	1.43	9.58	29.73	109.48	0.38	51.72	27.19	0	0
28/06/2020	71.38	1.57	9.67	30.24	161.65	1.30	48.39	24.61	0	0
29/06/2020	73.06	1.65	9.40	29.97	161.57	3.51	46.17	24.75	6	7
30/06/2020	70.02	2.11	9.63	30.56	161.65	0.09	48.89	25.22	0	2
01/07/2020	70.87	1.58	9.33	29.96	161.03	2.40	46.56	25.67	0	2
02/07/2020	75.17	1.96	9.28	29.46	213.06	4.27	45.67	26.93	0	6
03/07/2020	78.44	1.90	9.29	28.51	109.67	9.73	49.78	26.82	0	1
04/07/2020	76.96	1.37	8.96	28.74	110.75	1.23	55.67	33.29	1	5
05/07/2020	70.95	1.46	9.42	29.97	161.57	0.84	51.83	27.93	5	5
06/07/2020	70.01	1.45	9.81	30.84	161.02	6.66	49.39	26.63	5	5
07/07/2020	79.50	1.26	9.30	29.42	110.45	5.37	54.06	29.22	0	0
08/07/2020	73.57	1.37	9.60	30.53	110.48	2.83	54.00	28.18	1	2
09/07/2020	70.50	1.42	9.69	31.10	161.43	0.13	53.83	27.18	3	5
10/07/2020	69.48	1.46	9.63	31.03	109.45	1.59	51.50	27.54	0	0
11/07/2020	71.87	1.40	9.34	30.64	109.31	1.13	53.67	29.11	12	14
12/07/2020	73.31	1.33	9.32	30.35	161.09	1.59	54.28	26.61	1	1
13/07/2020	77.31	1.30	8.90	29.06	109.34	25.45	47.39	24.36	2	3
14/07/2020	80.56	1.05	9.02	28.42	109.78	14.99	53.39	27.96	1	7
15/07/2020	78.61	1.19	9.25	28.81	115.01	10.76	56.94	29.14	3	5
16/07/2020	77.69	1.24	9.33	29.00	161.34	7.23	51.61	28.61	1	4
17/07/2020	75.30	1.29	9.37	29.58	109.64	3.47	52.61	28.32	0	3
18/07/2020	72.91	1.24	9.63	30.66	110.21	1.83	59.94	31.96	6	7
19/07/2020	75.36	1.37	9.40	30.36	109.52	4.24	49.06	23.21	3	3
20/07/2020	77.75	0.91	9.28	29.49	109.74	11.50	63.22	33.82	1	1
21/07/2020	81.76	0.99	8.92	28.40	110.69	5.80	61.83	33.32	2	5
22/07/2020	78.52	0.83	9.15	29.16	110.34	1.54	64.89	33.79	2	6

23/07/2020	74.57	1.08	9.64	30.12	110.41	4.50	59.06	29.07	2	8
24/07/2020	78.44	1.20	9.00	28.98	110.42	14.87	59.39	30.52	7	10
25/07/2020	80.71	1.09	9.21	28.49	110.19	6.93	59.33	30.15	2	3
26/07/2020	74.81	1.09	9.59	29.88	109.66	5.91	56.06	25.64	5	9
27/07/2020	71.38	1.37	9.59	30.68	114.79	0.71	45.61	21.14	3	4
28/07/2020	78.20	0.97	9.38	29.27	109.95	7.78	45.06	22.26	0	2
29/07/2020	74.77	1.28	9.34	29.30	110.88	7.65	48.11	26.89	0	1
30/07/2020	69.73	1.37	9.60	30.37	109.25	0.51	45.00	25.32	0	6
31/07/2020	68.67	1.68	9.51	30.91	212.20	0.36	47.11	24.14	2	6
01/08/2020	71.36	2.20	9.05	30.47	263.12	0.83	40.44	22.61	0	2
02/08/2020	81.28	2.58	8.18	28.06	419.63	3.34	38.39	19.21	1	5
03/08/2020	79.13	2.20	8.72	28.51	387.41	2.58	39.39	19.36	1	3
04/08/2020	85.13	2.15	8.00	27.72	211.60	20.99	42.72	22.89	0	1
05/08/2020	80.67	1.41	8.87	28.51	108.74	3.75	44.78	24.70	0	7
06/08/2020	76.03	1.33	9.23	29.58	109.35	2.30	55.17	30.00	0	2
07/08/2020	85.39	0.95	8.53	27.64	109.15	2.32	57.61	32.22	1	15
08/08/2020	80.00	1.31	9.17	28.74	161.02	4.99	53.39	27.00	0	3
09/08/2020	76.67	1.70	9.38	29.91	160.87	4.56	46.83	21.96	1	3
10/08/2020	75.62	1.65	9.30	29.48	109.53	1.36	44.78	22.93	0	0
11/08/2020	77.80	1.77	9.26	29.49	109.81	2.25	50.67	26.54	0	0
12/08/2020	81.55	1.40	8.80	28.65	109.80	11.52	46.67	23.26	1	5
13/08/2020	82.53	1.65	8.63	28.01	109.61	6.26	52.89	25.56	2	3
14/08/2020	86.60	1.50	8.23	26.53	110.64	11.66	60.06	30.07	0	17
15/08/2020	85.47	1.11	8.62	27.32	111.18	8.55	64.50	33.22	0	0
16/08/2020	78.83	1.17	9.05	29.15	110.34	2.35	60.33	31.68	1	1
17/08/2020	79.15	1.16	8.80	29.06	109.56	4.51	64.83	35.50	0	1
18/08/2020	76.09	1.03	8.90	29.22	109.22	3.74	69.44	42.39	3	3
19/08/2020	75.60	1.13	9.10	29.61	109.02	6.53	67.22	38.39	1	1
20/08/2020	78.40	0.87	8.79	29.16	110.05	4.60	76.28	42.67	4	7
21/08/2020	79.40	1.27	8.51	28.66	111.16	4.86	64.50	35.59	0	1
22/08/2020	77.90	1.53	8.96	28.90	110.15	5.21	55.72	28.59	0	0
23/08/2020	78.17	1.51	9.11	28.67	110.87	10.54	52.17	25.96	1	5
24/08/2020	71.09	1.18	9.42	30.18	111.48	6.54	59.17	32.89	1	2
25/08/2020	66.13	1.32	9.92	30.91	110.87	0.00	62.61	35.21	2	5
26/08/2020	62.44	1.63	9.66	31.22	109.84	0.00	59.61	33.29	1	1
27/08/2020	69.50	1.60	9.21	30.13	161.03	2.18	54.61	30.39	0	1
28/08/2020	76.65	1.34	9.03	28.88	160.41	14.38	57.78	32.39	2	6
29/08/2020	72.18	1.15	9.40	30.27	160.25	2.08	61.28	31.96	0	1
30/08/2020	76.38	0.91	9.18	30.04	160.48	4.12	65.61	35.89	0	0
31/08/2020	78.33	1.23	8.73	29.81	108.77	11.09	60.22	30.52	0	1
01/09/2020	84.57	0.66	8.46	27.66	110.52	41.21	48.94	28.67	0	5

02/09/2020	77.52	0.86	8.96	29.52	110.65	8.13	66.78	39.18	0	8
03/09/2020	76.18	0.89	8.99	29.98	110.42	2.91	62.17	35.64	1	2
04/09/2020	78.33	0.84	9.02	29.82	109.86	8.60	62.78	30.89	4	4
05/09/2020	79.20	0.88	8.94	29.78	109.44	12.87	58.56	27.32	0	7
06/09/2020	78.52	1.28	9.08	29.66	109.78	5.91	49.56	21.68	5	6
07/09/2020	79.73	1.08	9.11	29.10	110.18	7.07	46.94	21.32	1	1
08/09/2020	78.42	0.98	9.21	29.21	111.03	5.68	52.50	26.07	0	1
09/09/2020	78.52	0.74	9.18	29.54	110.21	3.01	66.17	34.00	0	1
10/09/2020	77.28	0.74	9.27	29.86	108.94	2.53	61.94	30.78	5	7
11/09/2020	76.69	1.14	9.31	29.98	110.17	1.62	56.44	25.93	3	7
12/09/2020	78.65	0.88	9.29	29.70	111.52	3.59	53.44	25.64	1	5
13/09/2020	80.79	0.94	9.19	29.42	111.71	3.04	50.89	23.61	2	7
14/09/2020	79.95	1.00	9.26	29.34	110.72	7.03	54.72	26.79	0	2
15/09/2020	79.72	1.03	9.33	29.17	110.57	9.02	56.22	28.43	1	5
16/09/2020	76.52	1.17	9.35	29.61	109.59	5.91	54.28	27.46	6	10
17/09/2020	75.64	1.20	9.08	30.17	212.64	1.36	53.50	27.79	0	0
18/09/2020	85.56	1.42	7.70	28.04	314.75	17.49	56.67	28.56	4	7
19/09/2020	89.75	2.42	7.47	26.98	263.57	25.62	42.33	18.75	3	3
20/09/2020	89.05	1.42	8.23	27.14	110.91	5.45	39.95	20.30	5	6
21/09/2020	82.16	0.80	8.98	28.80	111.64	5.27	58.43	32.86	0	0
22/09/2020	83.32	0.63	8.90	29.10	111.22	3.80	63.70	39.36	0	5
23/09/2020	85.63	0.99	7.98	28.00	111.59	16.32	74.95	45.39	0	3
24/09/2020	83.07	1.10	8.82	27.97	112.32	10.68	46.56	26.48	2	2
25/09/2020	75.68	1.24	9.50	29.49	111.40	1.14	45.91	27.70	0	3
26/09/2020	73.31	1.27	9.47	30.23	110.59	0.27	46.58	26.57	0	3
27/09/2020	76.07	1.42	9.19	29.34	108.99	21.60	46.53	24.41	0	1
28/09/2020	88.04	0.84	8.35	27.25	108.52	23.96	52.27	26.04	0	22
29/09/2020	84.98	1.24	8.71	27.96	109.24	10.38	46.44	22.48	4	14
30/09/2020	77.83	1.26	9.27	28.76	109.35	2.24	50.69	28.29	3	5
01/10/2020	78.18	1.18	9.57	29.07	114.71	7.33	52.56	27.00	0	5
02/10/2020	81.86	0.94	8.72	28.45	109.18	22.45	53.81	29.07	1	6
03/10/2020	87.32	0.77	8.46	27.47	110.48	17.07	61.50	27.86	0	8
04/10/2020	82.87	0.88	8.86	28.62	110.88	8.67	53.31	24.43	2	2
05/10/2020	82.40	0.64	8.71	28.57	110.85	8.97	61.25	33.32	0	5
06/10/2020	83.89	0.83	9.01	27.98	110.95	10.21	70.31	37.29	3	10
07/10/2020	80.80	1.28	8.73	28.52	110.02	4.95	66.38	40.79	2	15
08/10/2020	91.80	1.64	6.85	25.19	109.37	19.60	52.40	26.04	1	7
09/10/2020	87.48	1.64	7.52	25.66	110.06	8.93	52.00	25.96	2	6
10/10/2020	88.87	1.03	7.21	25.17	110.06	11.08	56.63	28.44	2	6
11/10/2020	84.66	0.98	8.08	25.86	109.06	2.41	64.31	34.70	0	2
12/10/2020	80.75	0.66	8.43	27.47	108.55	1.07	82.73	49.11	5	5

13/10/2020	89.65	0.98	7.06	25.15	109.76	12.04	72.13	36.44	1	2
14/10/2020	81.10	0.63	8.45	27.92	108.85	4.07	68.13	35.67	0	9
15/10/2020	79.34	1.00	9.00	28.75	160.78	7.53	57.87	36.37	0	13
16/10/2020	92.03	0.78	7.21	26.47	160.86	39.70	54.81	29.85	1	4
17/10/2020	92.69	1.18	7.10	24.76	108.88	25.37	43.94	19.96	7	10
18/10/2020	91.92	1.07	7.31	23.93	110.92	10.40	44.67	21.78	2	7
19/10/2020	87.40	0.76	7.96	25.15	111.91	1.24	60.60	36.33	2	5
20/10/2020	83.31	1.18	8.39	25.81	60.38	0.15	60.53	38.25	3	9
21/10/2020	81.16	1.14	8.87	26.62	60.26	0.39	61.60	36.04	5	9
22/10/2020	74.73	1.07	9.13	27.30	59.67	0.13	72.33	45.11	1	10
23/10/2020	69.90	0.91	8.90	27.26	60.13	0.03	83.73	53.64	2	8
24/10/2020	67.65	1.03	8.62	27.02	60.49	0.00	94.07	67.18	3	4
25/10/2020	68.82	0.85	8.58	27.72	60.31	0.15	110.40	77.26	1	5
26/10/2020	77.29	0.73	7.67	27.58	111.65	0.19	118.27	80.77	1	7
27/10/2020	81.29	0.95	8.36	26.80	111.78	0.15	89.87	53.86	0	3
28/10/2020	78.33	1.17	8.21	28.00	110.65	0.03	83.87	50.11	7	12
29/10/2020	87.90	1.16	7.56	25.90	110.57	16.99	71.87	36.93	1	5
30/10/2020	85.67	0.98	7.82	26.95	60.20	4.29	57.75	30.88	1	12
31/10/2020	86.93	0.94	8.31	27.22	8.78	14.06	66.00	37.04	0	5
01/11/2020	82.79	1.03	8.75	27.63	8.93	5.49	61.00	36.89	1	4
02/11/2020	74.90	1.14	8.96	28.82	9.21	0.13	73.67	48.93	2	3
03/11/2020	70.73	1.50	8.99	28.67	9.42	0.00	74.25	47.46	1	10
04/11/2020	72.92	1.13	8.70	27.96	9.49	0.00	83.19	53.46	2	7
05/11/2020	72.18	0.99	8.98	28.19	9.45	0.00	78.69	51.59	1	6
06/11/2020	72.64	1.15	8.81	28.46	9.51	0.79	84.94	54.14	2	8
07/11/2020	76.33	1.67	7.90	27.12	10.39	0.40	72.25	43.54	1	12
08/11/2020	70.07	1.59	8.07	27.39	10.49	0.51	99.69	58.54	1	7
09/11/2020	66.28	1.63	8.52	27.15	9.75	0.00	96.38	58.21	0	3
10/11/2020	61.51	1.86	8.54	26.82	9.06	0.00	97.75	64.31	1	4
11/11/2020	72.07	2.18	8.25	24.87	10.63	0.76	91.31	58.54	2	3
12/11/2020	78.18	1.70	8.12	24.70	10.89	0.74	80.44	47.32	2	5
13/11/2020	62.70	1.45	8.40	26.40	9.38	0.17	96.00	63.54	1	9
14/11/2020	61.22	1.12	8.51	25.90	8.74	0.00	108.88	78.29	2	5
15/11/2020	64.23	1.06	8.68	27.43	9.01	0.00	118.81	82.21	3	8
16/11/2020	70.01	0.96	8.70	28.20	9.17	0.00	79.25	51.75	0	1
17/11/2020	72.10	0.61	9.07	28.72	60.21	0.00	86.63	63.04	0	3
18/11/2020	72.08	0.56	9.27	29.29	62.91	0.00	90.88	66.07	1	2
19/11/2020	70.39	0.63	8.44	29.81	111.31	0.00	105.81	75.29	6	8
20/11/2020	74.03	0.71	8.54	29.46	111.91	2.46	103.50	68.46	2	4
21/11/2020	78.08	0.71	8.26	28.54	63.31	7.22	86.94	54.68	5	10
22/11/2020	81.52	0.85	8.23	27.80	9.11	17.06	77.19	44.63	5	11

23/11/2020	73.80	1.13	8.71	28.62	9.21	2.07	88.56	59.04	3	7
24/11/2020	70.77	1.47	9.23	28.48	9.17	0.00	67.06	49.71	0	2
25/11/2020	70.40	1.40	9.01	28.35	9.02	0.00	70.63	52.07	3	4
26/11/2020	67.00	1.61	9.20	28.53	9.70	0.00	67.63	48.89	2	16
27/11/2020	67.25	2.17	9.63	27.90	10.71	0.00	60.69	41.54	5	19
28/11/2020	64.61	1.99	9.15	27.54	10.66	0.00	72.31	51.43	2	5
29/11/2020	65.44	1.92	9.20	27.35	10.44	0.00	69.13	44.86	3	11
30/11/2020	63.85	2.23	9.36	26.90	10.74	0.00	61.93	41.43	7	21
01/12/2020	65.36	1.97	9.01	26.89	11.07	0.00	66.25	42.82	2	10
02/12/2020	67.14	1.74	9.58	27.30	9.95	0.00	66.06	41.75	6	18
03/12/2020	65.70	2.12	9.78	26.74	10.32	0.00	66.06	43.93	4	13
04/12/2020	61.50	2.25	8.93	25.16	10.94	0.00	77.00	50.33	4	14
05/12/2020	62.36	2.11	9.27	23.87	11.99	0.00	77.81	50.96	4	19
06/12/2020	66.14	1.23	9.07	24.08	11.55	0.00	86.60	57.04	8	14
07/12/2020	64.06	1.28	8.74	25.66	11.46	0.00	97.88	68.56	2	21
08/12/2020	66.30	1.52	8.93	25.90	10.83	0.00	91.69	63.07	6	19
09/12/2020	65.72	1.17	8.35	25.07	9.53	0.00	97.44	65.71	1	25
10/12/2020	63.96	1.10	8.07	25.25	9.34	0.00	117.31	80.86	2	18
11/12/2020	65.36	0.94	8.51	26.89	8.49	0.00	126.00	86.00	3	11
12/12/2020	67.17	0.71	8.48	28.35	63.24	0.00	121.00	86.68	1	12
13/12/2020	69.37	0.74	7.92	28.74	111.55	0.00	126.06	87.79	2	17
14/12/2020	76.10	0.52	7.14	28.19	60.24	0.50	141.88	95.54	5	28
15/12/2020	71.22	0.99	7.80	28.68	9.26	0.00	124.06	96.79	0	9
16/12/2020	70.81	1.32	8.38	28.08	9.81	0.01	95.75	70.00	4	15
17/12/2020	67.22	1.33	8.88	27.13	9.39	0.00	75.25	63.68	6	20
18/12/2020	64.53	1.54	8.99	27.19	9.75	0.00	80.83	62.00	2	16
19/12/2020	62.60	2.00	9.15	25.90	11.09	0.00	71.72	53.96	13	34
20/12/2020	60.28	2.38	9.22	23.77	12.18	0.00	71.82	50.71	25	576
21/12/2020	59.68	2.17	8.97	22.59	12.02	0.00	78.47	54.21	17	382
22/12/2020	60.94	1.65	8.39	22.78	10.40	0.00	97.39	67.43	12	427
23/12/2020	63.07	1.34	8.47	24.09	8.79	0.00	116.06	75.21	22	46
24/12/2020	61.34	1.23	8.51	26.63	60.40	0.00	114.56	77.04	45	67
25/12/2020	67.33	1.23	8.43	26.55	61.07	0.00	102.94	70.54	26	81
26/12/2020	69.90	0.77	8.55	27.70	8.74	0.00	103.44	69.75	41	110
27/12/2020	69.89	0.72	8.53	28.60	63.37	0.00	104.67	64.18	42	121
28/12/2020	70.95	0.73	8.15	28.55	112.25	0.00	90.00	52.57	52	144
29/12/2020	69.20	0.63	7.93	28.53	63.01	0.00	97.11	59.86	44	155
30/12/2020	56.87	2.65	8.58	27.69	9.14	0.00	95.56	62.86	32	250
31/12/2020	57.07	2.69	8.88	23.62	12.20	0.00	69.67	46.71	37	194
01/01/2021	58.61	2.27	8.87	22.26	12.91	0.00	75.56	44.90	24	279
02/01/2021	58.46	1.57	8.80	22.83	11.79	0.00	83.24	49.90	45	216

03/01/2021	58.13	1.49	8.27	24.92	10.31	0.00	110.24	65.31	46	315
04/01/2021	61.80	1.60	8.65	25.79	10.39	0.00	100.11	63.72	153	745
05/01/2021	62.19	1.07	8.63	27.38	9.74	0.00	102.61	73.29	81	527
06/01/2021	63.58	1.10	8.38	27.69	60.73	0.00	107.39	75.79	121	365
07/01/2021	64.00	1.11	8.60	27.98	63.19	0.00	95.39	67.48	120	305
08/01/2021	62.97	2.21	8.86	26.62	60.98	0.00	79.44	53.59	91	205
09/01/2021	60.81	1.88	8.95	23.94	10.13	0.00	68.67	51.24	124	212
10/01/2021	60.88	1.46	8.75	24.39	9.97	0.00	86.53	59.07	73	245
11/01/2021	57.05	2.68	8.73	23.96	12.01	0.00	91.00	61.66	122	249
12/01/2021	53.18	2.44	8.22	20.34	13.71	0.00	89.47	76.00	74	287
13/01/2021	57.53	1.01	8.08	20.57	11.89	0.00	122.50	90.00	74	157
14/01/2021	60.87	0.77	7.52	22.78	9.76	0.00	147.44	117.89	49	271
15/01/2021	62.80	0.66	6.78	24.38	60.34	0.00	153.17	126.83	56	188
16/01/2021	68.40	0.68	6.60	25.40	60.47	0.00	146.33	103.03	55	230
17/01/2021	62.87	1.71	6.83	26.05	10.91	0.00	127.17	81.46	134	374
18/01/2021	58.63	2.30	8.09	22.80	12.29	0.00	92.94	67.31	27	369
19/01/2021	59.87	1.33	7.77	21.70	11.24	0.00	124.22	83.90	35	171
20/01/2021	59.35	0.62	7.55	22.86	10.06	0.00	144.39	106.45	39	59
21/01/2021	59.59	0.60	7.02	23.93	8.65	0.00	147.44	119.54	52	142
22/01/2021	60.83	0.51	6.84	24.98	8.36	0.00	159.28	137.31	67	309
23/01/2021	62.46	0.86	7.11	26.41	60.49	0.00	148.17	107.14	52	198
24/01/2021	67.83	0.75	7.48	27.02	8.41	0.00	105.00	62.10	96	198
25/01/2021	68.39	0.74	7.47	27.48	8.53	0.00	91.50	56.89	53	187
26/01/2021	67.50	0.95	7.50	28.09	59.99	0.00	99.33	66.21	147	959
27/01/2021	68.16	1.08	7.35	28.29	60.99	0.00	104.61	67.93	39	819
28/01/2021	65.83	1.17	7.44	28.79	9.16	0.00	106.89	70.14	16	756
29/01/2021	61.22	1.63	7.94	27.00	10.55	0.00	110.89	78.00	220	802
30/01/2021	60.88	1.40	7.35	26.65	10.39	0.00	117.72	84.03	93	930
31/01/2021	61.29	0.97	7.61	26.50	10.91	0.00	119.50	85.03	83	829
01/02/2021	61.13	0.84	7.76	27.57	10.44	0.00	139.39	111.34	112	836
02/02/2021	60.72	0.82	7.65	28.40	10.15	0.00	130.82	103.66	94	836
03/02/2021	60.77	1.20	7.54	28.01	10.17	0.00	129.59	100.72	107	795
04/02/2021	58.87	1.35	7.70	26.86	10.97	0.00	124.59	93.54	222	809
05/02/2021	55.95	1.00	7.71	27.00	10.03	0.00	119.71	95.97	31	586
06/02/2021	58.00	0.95	7.90	27.31	8.66	0.00	120.53	95.41	54	490
07/02/2021	62.79	0.98	7.42	28.06	60.98	0.00	135.76	96.55	51	237
08/02/2021	72.17	1.50	7.12	28.16	60.39	0.49	103.94	66.07	65	186
09/02/2021	80.63	1.50	6.87	25.95	60.90	13.04	101.12	68.54	73	189
10/02/2021	61.07	1.68	9.07	27.63	61.68	10.81	84.59	60.39	69	157
11/02/2021	72.69	0.74	7.70	27.15	40.26	4.87	92.71	67.24	57	201
12/02/2021	65.58	1.21	7.53	27.80	19.20	0.48	104.59	71.61	67	175

13/02/2021	67.83	1.41	7.92	26.05	9.16	0.00	112.88	68.66	29	126
14/02/2021	68.16	1.59	7.76	27.97	9.16	0.00	94.35	53.00	81	166
15/02/2021	68.21	1.55	7.48	28.09	60.43	0.00	88.88	53.93	87	143
16/02/2021	57.16	2.46	8.19	28.76	60.16	0.00	89.65	55.61	38	72
17/02/2021	51.80	2.94	8.78	28.46	9.63	1.21	101.71	65.24	75	175
18/02/2021	64.53	0.57	6.80	27.16	12.60	1.65	116.00	79.10	46	150
19/02/2021	63.85	1.27	7.36	27.48	13.78	0.00	104.59	71.21	58	130
20/02/2021	64.53	1.30	7.59	27.80	19.20	0.48	107.29	76.21	41	82
21/02/2021	64.56	1.72	7.28	27.30	9.72	0.00	138.88	97.32	60	92
22/02/2021	60.57	1.32	7.42	27.63	9.18	0.00	134.65	88.21	45	89
23/02/2021	57.04	1.27	7.26	28.81	60.26	0.00	111.18	75.57	54	95
24/02/2021	60.17	1.28	6.80	28.67	111.61	0.00	118.25	74.46	56	93
25/02/2021	66.82	1.76	6.65	29.24	109.95	0.00	121.69	78.90	38	72
26/02/2021	65.60	1.46	7.33	29.33	161.45	0.00	131.81	80.24	20	45
27/02/2021	67.73	1.67	7.94	28.89	109.57	0.00	130.13	80.93	40	72
28/02/2021	75.61	1.91	8.11	28.62	111.56	0.00	126.00	74.79	39	70
01/03/2021	70.88	1.57	8.53	29.20	8.93	0.00	89.88	53.14	22	80
02/03/2021	67.98	1.70	8.44	29.94	61.08	0.00	67.59	37.21	22	42
03/03/2021	66.91	1.50	8.67	28.59	9.47	2.11	56.94	34.24	10	35
04/03/2021	65.06	1.69	8.20	29.52	9.35	0.04	71.06	43.39	19	54
05/03/2021	67.60	1.49	8.30	29.65	9.19	0.81	69.47	42.93	26	79
06/03/2021	67.55	1.33	8.13	30.09	9.08	0.00	72.41	45.52	28	64
07/03/2021	67.48	1.23	7.78	30.28	8.87	0.00	74.19	44.97	19	65
08/03/2021	67.22	1.02	7.79	29.65	9.73	1.83	84.82	50.52	28	71
09/03/2021	66.05	1.38	7.60	29.55	9.49	0.75	84.24	49.79	12	60
10/03/2021	68.73	1.46	7.77	29.65	9.37	0.88	94.71	57.62	20	39
11/03/2021	69.40	1.55	7.57	30.02	9.29	0.09	104.76	62.21	13	58
12/03/2021	68.51	1.83	7.68	30.31	9.46	0.03	100.35	60.93	17	81
13/03/2021	68.90	1.70	7.29	30.06	8.66	0.00	95.76	56.97	30	78
14/03/2021	70.12	1.77	7.37	29.83	59.94	0.00	90.18	54.28	17	170
15/03/2021	68.49	1.73	7.09	29.96	111.73	0.00	86.88	52.38	11	78
16/03/2021	66.81	1.83	7.23	30.00	111.53	0.64	88.59	54.83	21	149
17/03/2021	66.95	1.85	7.23	30.06	111.15	0.00	95.29	57.34	54	248
18/03/2021	68.38	1.80	7.70	30.68	110.84	0.00	109.06	67.34	56	92
19/03/2021	70.72	2.03	7.89	30.93	110.09	0.00	101.71	62.54	48	100
20/03/2021	60.12	1.50	8.50	30.96	109.14	0.00	96.94	59.59	75	119
21/03/2021	59.40	1.22	8.21	30.95	110.22	1.08	84.65	49.72	31	90
22/03/2021	64.54	1.50	7.67	28.03	9.57	5.55	103.06	66.10	27	73
23/03/2021	67.85	1.87	7.71	29.15	61.68	0.03	98.71	70.17	44	401
24/03/2021	67.80	1.82	8.19	30.85	111.82	0.00	122.82	83.79	50	69
25/03/2021	66.49	1.97	8.27	30.98	111.29	0.00	117.12	71.76	70	97

26/03/2021	65.20	1.83	8.11	30.75	111.43	1.77	75.94	44.62	50	134
27/03/2021	63.30	2.12	8.20	30.87	110.30	0.00	67.35	42.29	44	80
28/03/2021	63.26	1.85	7.92	31.39	109.94	0.00	67.59	40.17	40	77
29/03/2021	65.63	1.92	7.87	31.39	161.22	0.00	66.44	39.76	20	39
30/03/2021	67.21	1.84	8.54	31.30	212.80	0.00	81.40	49.83	27	48
31/03/2021	69.45	1.67	8.77	31.47	212.18	0.09	92.67	54.31	17	42
01/04/2021	83.15	1.47	8.03	31.22	160.39	0.00	77.50	43.00	12	26
02/04/2021	83.40	0.94	7.30	31.54	161.03	0.00	53.56	28.29	38	58
03/04/2021	83.90	1.09	7.26	31.24	108.83	2.26	62.33	33.10	57	84
04/04/2021	76.84	1.10	7.97	27.16	111.58	20.80	61.17	33.83	55	96
05/04/2021	72.66	1.10	8.37	27.08	60.26	3.46	84.06	51.28	63	194
06/04/2021	70.49	1.20	8.52	27.14	8.81	5.65	90.67	54.86	62	250
07/04/2021	73.69	1.10	8.31	28.14	8.50	6.20	79.22	50.21	148	334
08/04/2021	76.57	1.08	7.71	29.37	111.87	0.84	87.11	50.62	163	405
09/04/2021	74.76	0.97	8.11	30.00	111.86	0.93	80.44	44.14	291	559
10/04/2021	72.01	1.34	8.60	29.40	60.73	6.56	89.00	53.43	313	789
11/04/2021	75.43	1.29	8.31	28.63	9.33	7.22	84.94	47.00	166	967
12/04/2021	70.12	1.06	8.97	29.43	9.01	3.40	82.11	45.52	242	984
13/04/2021	73.01	1.48	8.67	29.83	60.61	4.39	70.67	34.00	314	964
14/04/2021	72.83	1.00	8.65	28.86	8.28	4.38	63.17	30.07	499	1338
15/04/2021	74.03	1.25	8.72	29.98	111.48	0.83	70.11	36.07	679	1546
16/04/2021	76.83	0.94	9.35	29.96	111.87	0.96	62.39	31.24	438	1581
17/04/2021	72.12	1.00	9.33	30.01	8.43	0.86	64.28	33.28	342	1547
18/04/2021	74.34	0.87	9.18	29.11	112.47	16.71	61.67	32.31	500	1766
19/04/2021	70.22	1.20	9.10	28.81	111.16	6.36	48.89	25.83	520	1391
20/04/2021	69.68	1.54	8.67	30.16	110.74	2.73	53.89	33.17	599	1443
21/04/2021	70.76	1.40	8.38	30.01	111.09	3.24	61.72	35.41	614	1458
22/04/2021	73.53	1.17	8.37	31.07	110.25	0.95	77.22	44.38	714	1470
23/04/2021	76.08	0.99	8.47	31.07	109.12	4.98	73.50	43.03	963	2070
24/04/2021	86.62	0.91	7.91	30.93	109.38	1.11	74.00	44.59	1160	2839
25/04/2021	80.53	0.94	8.26	30.03	110.52	5.05	75.11	43.25	1276	2438
26/04/2021	84.20	0.90	8.32	29.40	110.75	7.42	70.89	42.11	976	2048
27/04/2021	80.94	0.66	8.47	27.48	60.29	20.68	67.89	41.46	1193	2179
28/04/2021	80.63	0.62	8.50	28.31	60.78	9.41	78.00	47.57	1252	2012
29/04/2021	78.07	0.80	8.62	27.41	60.92	15.71	62.06	34.52	1019	1871
30/04/2021	73.83	0.67	9.06	28.05	60.60	4.06	67.56	39.52	817	1583
01/05/2021	71.86	0.93	9.10	28.66	60.57	5.89	61.22	31.34	1147	1891
02/05/2021	70.04	0.87	9.08	29.03	112.56	7.52	72.33	41.07	1151	1940
03/05/2021	82.67	1.12	8.50	30.18	110.98	4.31	71.89	46.25	1276	2041
04/05/2021	76.27	0.85	9.19	30.20	110.53	6.17	82.39	47.55	827	1763
05/05/2021	76.31	0.90	8.89	31.04	110.61	1.85	80.17	51.24	1010	2112

06/05/2021	75.09	0.81	9.10	26.80	9.22	10.67	66.67	41.24	1368	1911
07/05/2021	70.60	1.17	9.43	28.50	8.27	8.92	55.11	35.34	1381	2044
08/05/2021	70.83	1.44	9.60	28.81	112.05	7.13	74.56	47.21	1569	2419
09/05/2021	69.60	1.25	9.43	29.59	110.89	0.43	65.67	36.14	1262	2101
10/05/2021	70.67	1.44	9.80	31.06	110.34	0.55	65.28	34.21	482	1630
11/05/2021	68.12	1.44	9.76	31.30	109.61	0.31	60.89	32.48	403	1919
12/05/2021	73.66	1.23	9.50	31.69	161.27	0.33	56.94	29.48	406	1983
13/05/2021	73.24	1.20	9.51	31.53	161.03	0.36	53.89	28.93	3108	4887
14/05/2021	69.48	1.08	9.67	32.13	161.11	0.00	54.83	29.48	606	2256
15/05/2021	72.71	1.38	9.43	31.87	115.16	0.27	58.22	29.90	1265	3095
16/05/2021	71.00	1.45	9.25	31.12	109.48	4.45	54.33	26.29	410	2302
17/05/2021	72.08	1.25	9.73	31.98	109.71	0.55	55.22	29.67	486	9635
18/05/2021	67.86	1.47	9.54	31.60	110.01	0.08	49.06	26.28	1764	2473
19/05/2021	66.71	1.67	9.72	31.24	109.33	20.84	54.78	30.76	1818	3394
20/05/2021	64.26	2.03	9.89	31.12	109.62	9.70	54.83	28.93	1589	2636
21/05/2021	65.90	1.60	9.62	31.65	109.08	0.61	45.44	25.31	2008	3481
22/05/2021	73.32	1.23	9.43	31.20	161.19	1.00	41.50	23.79	1466	3052
23/05/2021	71.04	1.29	9.40	31.61	161.52	0.38	41.67	23.62	1178	3382
24/05/2021	74.39	1.22	8.89	31.76	109.22	0.49	46.39	27.28	1161	2713
25/05/2021	83.47	1.10	8.82	30.19	109.15	4.53	50.33	30.10	1740	3226
26/05/2021	78.83	1.36	9.16	30.59	161.71	0.84	55.22	30.89	697	2455
27/05/2021	74.40	1.30	9.48	29.85	109.45	20.96	59.61	32.03	1550	3323
28/05/2021	70.95	1.44	9.88	28.57	110.96	8.95	53.11	28.93	1488	3759
29/05/2021	67.37	1.29	9.89	29.51	110.48	6.44	45.56	24.62	2896	4803
30/05/2021	67.44	1.20	9.70	30.31	109.57	2.89	39.06	20.62	2177	4528
31/05/2021	67.51	1.57	9.91	31.16	108.93	0.74	47.56	24.54	581	5485
01/06/2021	67.47	1.57	9.80	31.89	109.16	1.79	54.00	27.69	470	2230
02/06/2021	68.91	1.77	9.23	32.09	109.28	1.33	55.33	29.24	1365	3440
03/06/2021	69.29	1.68	9.49	32.34	109.95	0.00	53.00	27.10	1142	3886
04/06/2021	70.49	2.07	9.32	32.38	162.08	0.21	52.67	29.62	1057	2631
05/06/2021	75.50	1.89	9.05	31.50	109.73	1.69	53.11	29.31	1271	2817
06/06/2021	77.16	1.55	9.16	30.79	161.65	3.94	49.50	24.75	1324	2671
07/06/2021	74.22	1.69	9.28	30.17	161.57	0.50	44.00	23.72	1126	2419
08/06/2021	74.14	1.42	9.24	29.18	109.19	4.00	44.06	23.93	1357	2662
09/06/2021	74.93	1.08	9.05	29.02	109.13	4.48	41.22	23.55	1398	2680
10/06/2021	80.21	1.37	8.89	29.23	108.36	2.73	40.17	22.97	1310	2310
11/06/2021	78.50	1.33	8.77	29.03	160.82	2.41	48.50	28.72	1095	2290
12/06/2021	81.73	1.06	8.77	29.29	161.49	2.58	59.06	36.00	1507	3277
13/06/2021	76.31	1.39	9.33	28.23	109.33	5.91	60.56	36.66	1397	2804
14/06/2021	74.18	1.42	9.20	28.63	109.45	4.56	54.06	31.31	1329	3355
15/06/2021	70.22	1.33	9.85	27.78	109.81	3.93	49.50	27.00	1459	3000

16/06/2021	68.20	1.56	9.71	29.16	110.94	2.08	44.61	24.71	1115	2331
17/06/2021	68.65	1.60	9.62	29.35	112.47	1.61	48.33	26.38	1443	3129
18/06/2021	66.94	1.74	9.79	30.31	112.48	0.98	51.33	29.89	1347	3058
19/06/2021	68.05	1.64	9.54	30.75	111.56	3.75	50.83	28.69	2111	3667
20/06/2021	69.73	1.79	9.37	30.70	109.95	6.26	53.83	31.72	1510	3682
21/06/2021	68.29	1.58	9.54	30.88	161.77	2.58	70.72	50.07	1617	3175
22/06/2021	70.49	1.48	9.62	30.76	162.82	1.83	78.44	59.96	1708	4059
23/06/2021	70.83	1.71	9.62	30.40	110.88	1.14	65.72	39.31	1733	3174
24/06/2021	73.04	1.55	9.53	30.41	109.62	0.43	49.39	28.45	1778	4108
25/06/2021	81.03	1.22	9.24	30.34	161.51	0.51	63.22	36.00	1590	3644
26/06/2021	76.32	1.30	9.01	30.62	110.33	0.77	56.22	31.07	2167	4161
27/06/2021	71.17	1.54	9.66	30.20	111.54	11.19	51.61	25.28	1710	3995
28/06/2021	71.10	1.53	9.53	28.36	111.27	12.35	51.22	28.59	2258	5406
29/06/2021	70.31	1.13	9.57	29.60	109.92	5.35	59.50	34.83	2357	4662
30/06/2021	67.09	1.25	10.01	30.39	109.91	0.56	51.44	27.97	2376	4786
01/07/2021	64.65	1.47	10.08	30.02	110.33	7.11	45.72	24.14	2698	5533
02/07/2021	66.30	1.25	9.70	30.38	110.99	1.85	48.33	27.41	3462	6087
03/07/2021	74.95	1.50	9.40	31.21	109.66	1.56	48.17	25.11	3326	6230
04/07/2021	77.60	1.34	9.21	32.01	161.17	0.00	50.28	24.96	2980	5916
05/07/2021	78.12	1.10	9.11	31.68	212.59	0.71	48.78	25.79	3403	6166
06/07/2021	82.97	0.98	9.01	29.70	160.26	6.10	50.94	26.10	2935	5420
07/07/2021	86.98	1.15	8.49	29.30	161.09	7.02	46.83	25.04	2982	6519
08/07/2021	85.98	1.31	8.57	29.00	161.01	12.12	49.83	25.10	3598	7058
09/07/2021	77.47	1.01	9.47	28.37	160.21	7.86	49.72	26.86	4879	9276
10/07/2021	76.52	0.86	9.53	27.70	160.62	26.95	51.89	25.28	4370	9326
11/07/2021	83.68	0.75	8.55	27.69	110.35	13.01	37.50	18.64	4714	9539
12/07/2021	84.04	0.89	8.62	29.17	111.14	5.36	42.28	22.04	4840	8656
13/07/2021	81.07	1.10	9.18	29.53	110.07	1.56	54.94	29.61	4450	8685
14/07/2021	80.47	1.32	9.04	27.90	109.46	15.46	64.72	35.52	4700	9317
15/07/2021	75.87	1.99	9.36	27.66	110.63	19.61	51.61	27.21	3885	9186
16/07/2021	78.63	2.27	8.77	28.44	111.64	5.99	49.78	27.21	4684	9692
17/07/2021	78.55	1.82	9.02	28.65	109.70	10.63	50.11	25.62	4788	10082
18/07/2021	79.20	2.04	9.23	29.09	160.88	3.21	39.33	21.55	4824	11397
19/07/2021	79.85	2.05	8.61	27.96	161.67	5.98	42.39	19.07	5833	11784
20/07/2021	84.47	1.43	8.58	28.59	109.27	2.18	39.74	19.55	5480	11305
21/07/2021	86.56	1.99	7.99	29.14	161.42	1.61	42.42	21.24	5602	13002
22/07/2021	85.10	1.80	8.36	28.92	161.48	3.45	54.47	32.83	6625	13655
23/07/2021	78.70	1.75	8.68	28.09	115.02	2.95	50.42	29.03	7760	14575
24/07/2021	78.42	1.78	9.24	27.67	161.12	7.47	55.16	33.38	7083	14260
25/07/2021	83.49	1.67	8.86	27.42	161.45	5.55	45.84	22.62	6028	15335
26/07/2021	77.50	1.56	9.02	28.68	169.56	3.30	49.11	27.03	7260	15376

27/07/2021	78.12	1.55	9.19	29.04	212.86	6.43	40.21	22.45	6701	14150
28/07/2021	76.36	1.94	9.22	28.78	160.56	9.13	40.37	21.83	8146	16533
29/07/2021	74.16	1.78	9.18	29.48	161.16	2.68	47.79	26.03	6952	17669
30/07/2021	75.40	1.69	9.01	29.58	109.53	3.99	51.89	28.72	8794	17345
31/07/2021	77.72	1.93	9.04	29.65	109.41	1.83	55.37	31.69	8573	18912
01/08/2021	76.75	1.71	9.07	29.72	161.29	2.36	46.53	25.86	9486	18027
02/08/2021	78.28	1.87	9.21	29.62	161.48	2.16	48.00	27.79	8684	17970
03/08/2021	78.99	1.72	9.11	28.61	109.65	1.47	43.89	24.15	8260	18901
04/08/2021	76.30	1.57	9.42	29.36	109.75	1.90	50.78	28.45	9172	20200
05/08/2021	73.18	1.56	9.56	29.38	109.42	3.20	52.72	29.79	9645	20920
06/08/2021	71.74	1.54	9.41	29.25	110.07	7.16	49.28	27.31	10390	21379
07/08/2021	73.74	1.54	9.34	29.85	111.19	1.68	52.39	27.00	10893	21838
08/08/2021	81.71	1.41	9.26	30.55	110.72	1.08	56.83	31.17	9598	19983
09/08/2021	79.75	1.67	9.63	30.76	161.62	1.00	54.06	30.14	10057	19603
10/08/2021	81.03	1.28	9.00	30.93	160.33	0.41	56.72	30.39	10495	19843
11/08/2021	82.26	1.43	9.40	29.18	110.21	8.32	48.56	25.22	7378	21038
12/08/2021	82.90	1.70	8.70	29.46	111.59	7.44	42.56	20.93	11736	22782
13/08/2021	79.68	1.07	8.92	28.88	110.01	10.59	44.94	20.59	13352	23418
14/08/2021	73.78	1.01	9.64	28.39	109.19	3.29	49.17	25.31	12924	22086
15/08/2021	70.24	1.12	9.70	28.12	111.72	2.64	42.83	20.07	11255	21882
16/08/2021	71.30	0.99	9.54	28.27	9.13	3.91	43.67	21.41	11346	21157
17/08/2021	77.11	0.78	9.32	29.63	7.93	1.17	57.78	31.79	10388	20128
18/08/2021	78.23	1.01	9.07	30.45	111.03	0.48	56.17	30.72	9783	20515
19/08/2021	74.85	1.28	9.38	30.27	110.37	5.66	58.94	31.10	8879	20902
20/08/2021	73.63	1.60	9.83	29.64	111.35	5.94	68.50	37.90	9405	19851
21/08/2021	75.88	1.47	9.46	29.18	111.74	7.70	61.39	31.38	9047	20571
22/08/2021	79.69	1.26	9.07	29.90	110.28	4.98	55.83	27.18	4681	19014
23/08/2021	85.21	1.14	8.59	30.12	109.43	2.04	49.72	24.75	8651	17491
24/08/2021	87.03	1.09	8.50	29.65	160.96	8.52	48.33	24.14	7305	17165
25/08/2021	86.33	1.02	8.57	28.80	160.59	11.49	52.39	27.21	9019	18417
26/08/2021	81.87	0.95	9.29	27.61	109.02	16.42	43.28	22.25	8461	18501
27/08/2021	81.85	0.87	9.03	26.91	109.80	21.44	49.50	25.38	8677	18702
28/08/2021	84.63	0.82	8.52	27.31	109.77	10.97	47.06	23.28	8606	17984
29/08/2021	85.25	0.66	8.44	28.27	110.43	17.07	37.56	18.89	7224	16536
30/08/2021	82.67	0.90	8.81	28.76	111.04	4.46	53.33	29.19	6992	15972
31/08/2021	79.77	1.23	8.92	28.02	111.68	14.25	65.06	32.71	6928	14666
01/09/2021	81.57	1.50	9.04	27.85	111.08	8.22	63.47	32.82	6532	14802
02/09/2021	82.67	1.06	9.00	28.33	110.22	8.62	55.53	28.43	6725	14956
03/09/2021	79.98	0.91	9.05	28.06	110.05	2.39	42.81	23.67	6119	14653
04/09/2021	87.40	1.07	8.63	27.81	110.35	9.96	48.88	25.48	5863	15942
05/09/2021	88.07	0.94	8.32	27.87	111.51	3.97	53.00	26.93	5630	15452

06/09/2021	87.78	1.07	8.68	28.67	111.92	4.14	61.67	33.68	6041	13988
07/09/2021	84.77	1.05	8.83	27.54	110.63	16.10	56.94	31.90	6302	13821
08/09/2021	80.83	1.48	9.00	27.43	161.08	29.31	50.94	25.46	5863	14176
09/09/2021	74.29	1.57	9.25	27.68	108.77	7.07	51.78	27.54	6222	16031
10/09/2021	74.77	1.56	9.39	27.89	110.21	7.34	48.44	24.89	6038	14403
11/09/2021	81.37	1.12	9.10	28.14	110.36	2.98	47.11	26.43	5925	15191
12/09/2021	86.01	0.76	8.64	29.43	108.82	0.29	51.11	28.00	5661	14029
13/09/2021	85.60	1.14	9.14	29.82	108.90	0.34	49.67	29.75	5061	12583
14/09/2021	78.73	0.90	9.27	28.42	110.32	1.66	60.22	33.21	4868	11786
15/09/2021	78.66	0.78	9.14	27.82	110.97	8.29	57.94	28.68	5298	13798
16/09/2021	83.06	0.83	8.73	27.75	111.24	16.93	50.06	25.38	5290	13897
17/09/2021	85.90	0.85	8.03	28.99	111.73	3.12	54.22	28.55	5509	14555
18/09/2021	87.38	1.08	8.11	29.33	111.79	5.03	68.72	38.34	5193	14109
19/09/2021	87.07	1.22	8.41	27.71	111.59	17.55	61.50	32.00	4839	13576
20/09/2021	82.43	0.95	8.87	27.39	110.26	34.63	63.44	31.96	4781	12709
21/09/2021	84.93	1.14	8.65	26.93	109.19	26.71	49.56	23.75	3907	10919
22/09/2021	91.50	1.20	7.49	27.10	109.28	22.62	47.83	22.21	4249	11252
23/09/2021	88.70	1.40	8.40	28.22	110.85	9.97	52.56	25.10	4316	13256
24/09/2021	82.43	0.83	9.03	27.72	110.36	7.76	47.83	25.86	3702	12697
25/09/2021	81.44	0.80	9.19	25.97	161.33	42.28	43.50	25.00	3975	11975
26/09/2021	76.97	0.85	9.15	26.32	110.11	17.68	39.89	19.93	3741	12353
27/09/2021	80.90	0.85	9.11	27.93	111.79	6.31	52.00	29.34	3128	10288
28/09/2021	77.86	1.30	9.33	28.55	111.93	1.63	63.83	39.82	2882	9489
29/09/2021	81.49	1.30	8.94	29.96	110.81	1.82	84.53	55.86	2697	10414
30/09/2021	79.87	0.94	9.05	29.56	111.44	2.92	74.88	40.50	3338	11646
01/10/2021	79.17	0.90	9.47	29.99	111.65	6.31	63.33	30.74	3309	11754
02/10/2021	76.88	1.42	8.69	28.45	112.06	15.11	56.56	27.64	2803	11375
03/10/2021	84.69	1.15	8.87	28.51	111.34	8.98	64.94	33.64	2872	10828
04/10/2021	79.91	0.75	9.30	28.90	110.44	6.67	68.22	37.86	2347	9930
05/10/2021	78.36	0.85	9.07	29.37	109.75	11.50	76.50	45.14	2767	9869
06/10/2021	79.66	1.32	8.79	27.93	160.89	29.59	57.06	28.93	2665	9866
07/10/2021	76.76	1.56	9.58	28.74	160.28	12.28	64.00	38.00	2442	11200
08/10/2021	82.41	1.23	8.96	29.47	160.47	2.62	87.11	50.43	3164	11140
09/10/2021	81.48	1.15	9.03	28.83	160.84	1.30	59.22	30.18	3105	10630
10/10/2021	80.12	1.25	8.88	29.93	212.31	0.07	47.88	24.41	2795	10817
11/10/2021	83.44	1.17	8.80	29.10	168.32	1.74	42.72	23.36	2628	10035
12/10/2021	88.96	0.96	8.41	28.96	159.71	10.28	46.94	25.03	2502	9445
13/10/2021	83.27	1.29	8.51	29.14	211.72	3.21	40.61	20.79	2298	10064
14/10/2021	90.54	0.91	7.66	28.49	211.67	11.95	47.22	25.04	2725	11276
15/10/2021	85.78	1.53	8.42	27.29	108.21	18.81	58.56	27.18	2504	10486
16/10/2021	85.00	0.68	9.16	28.04	110.89	18.61	53.11	31.41	2482	10648

17/10/2021	80.88	0.67	8.96	25.17	8.40	28.64	39.67	20.79	2191	10863
18/10/2021	80.93	0.98	8.61	25.01	9.07	6.33	44.06	25.00	2250	10111
19/10/2021	83.43	0.84	8.62	27.17	10.58	0.88	62.67	43.26	1952	9122
20/10/2021	81.05	1.53	8.93	28.93	9.98	0.85	76.71	48.66	2175	8918
21/10/2021	74.88	1.57	9.13	28.73	9.02	4.18	81.00	48.14	2074	9727
22/10/2021	72.64	1.47	9.65	27.69	9.76	10.10	65.28	39.34	2097	9810
23/10/2021	70.92	1.36	9.17	27.30	10.62	5.75	56.72	30.83	1980	9742
24/10/2021	71.77	1.37	9.08	27.73	10.14	2.90	53.17	31.48	1934	9351
25/10/2021	82.77	1.41	8.48	28.01	8.65	0.00	61.22	35.62	2547	8675
26/10/2021	83.63	0.89	8.27	28.41	60.92	0.00	75.61	45.48	1821	7706
27/10/2021	83.20	1.01	8.42	28.51	60.70	0.00	80.00	47.48	1787	8452
28/10/2021	84.58	0.88	8.92	26.61	61.12	3.84	82.83	48.24	2205	9658
29/10/2021	83.27	0.84	8.83	27.55	9.67	7.43	71.33	37.97	2007	8968
30/10/2021	81.95	1.07	8.78	28.07	10.01	7.29	78.83	47.11	1870	9224
31/10/2021	78.76	0.79	9.18	27.70	9.78	6.72	58.44	34.75	1770	8859
01/11/2021	77.24	0.82	9.02	27.97	9.46	13.55	62.06	39.70	1645	8165
02/11/2021	75.77	0.80	9.15	28.19	9.22	15.23	65.72	42.85	1552	7574
03/11/2021	80.36	0.81	9.12	28.74	8.47	1.22	64.83	43.11	826	7679
04/11/2021	79.62	0.89	9.04	29.10	60.66	0.74	72.56	50.39	1732	7980
05/11/2021	77.22	1.70	8.83	29.34	111.79	0.25	71.61	49.36	1629	8150
06/11/2021	75.73	1.59	9.60	28.60	110.47	3.54	84.78	52.64	1828	8467
07/11/2021	70.52	1.67	9.34	28.90	110.46	6.64	73.56	45.75	1678	7960
08/11/2021	71.63	1.23	9.08	28.33	59.91	3.47	71.39	44.37	1701	7592
09/11/2021	72.16	1.47	9.23	27.52	9.06	0.88	58.33	36.78	1557	6904
10/11/2021	73.99	1.59	9.02	27.27	9.39	0.01	68.89	43.89	1553	6978
11/11/2021	74.04	1.34	8.70	27.17	8.92	0.00	77.11	53.56	1610	7496
12/11/2021	72.78	1.09	8.96	27.65	8.97	0.00	81.22	56.08	1502	7305
13/11/2021	76.80	1.38	9.33	26.82	9.05	0.00	77.44	49.70	1484	7057
14/11/2021	74.31	1.26	9.15	27.10	61.22	1.63	100.00	53.31	1396	7079
15/11/2021	75.00	1.21	9.53	28.22	112.18	0.20	88.11	55.88	1510	6343
16/11/2021	74.93	0.90	9.32	28.23	60.13	0.55	75.28	47.44	1610	5947
17/11/2021	76.02	0.56	9.11	29.02	60.55	0.58	64.39	39.00	1511	6524
18/11/2021	76.94	0.68	8.89	28.75	111.97	0.25	67.11	42.96	1628	6901
19/11/2021	72.86	1.13	8.97	29.30	110.22	0.14	73.11	47.50	1650	6855
20/11/2021	77.81	1.97	8.90	29.37	110.64	0.08	83.22	51.93	1662	6595
21/11/2021	71.63	1.76	9.48	29.39	111.46	0.34	88.67	50.41	1621	7006
22/11/2021	69.31	1.24	9.17	29.59	111.97	1.23	84.00	52.42	1855	6428
23/11/2021	68.39	1.71	9.08	26.74	61.61	0.86	63.61	38.67	1195	5126
24/11/2021	65.16	2.07	9.09	26.55	9.83	0.13	60.89	39.67	1380	5857
25/11/2021	63.42	1.89	9.07	27.23	9.68	0.00	71.91	52.32	1619	6428
26/11/2021	64.79	1.80	8.91	27.08	9.58	0.00	78.45	58.14	2171	5126

27/11/2021	61.69	2.23	8.74	26.17	10.31	0.00	77.00	53.46	1794	5857
28/11/2021	62.29	2.00	8.67	26.52	9.92	0.00	81.86	55.32	1829	6335
29/11/2021	63.54	1.84	8.56	27.26	8.95	0.01	77.77	54.14	1283	6559
30/11/2021	60.94	1.73	9.04	26.67	9.33	0.00	78.55	58.07	1363	6073
01/12/2021	60.45	1.81	8.91	25.41	10.43	0.00	88.55	64.82	1392	5854
02/12/2021	58.13	1.40	8.81	24.51	12.29	0.00	88.09	64.07	1450	4753
03/12/2021	59.16	1.51	8.86	24.37	13.22	0.00	86.86	65.68	1420	4306
04/12/2021	57.61	1.27	8.78	23.15	13.08	0.00	92.64	68.07	1364	4886
05/12/2021	59.00	1.50	8.90	22.53	12.81	0.00	96.64	70.43	1273	4971
06/12/2021	67.00	1.53	9.28	24.03	11.79	0.00	99.32	72.11	1221	4912
07/12/2021	67.93	1.17	9.15	24.45	12.04	0.00	111.91	80.29	1327	5896
08/12/2021	65.06	1.09	9.06	24.52	12.46	0.00	106.50	77.39	1188	4704
09/12/2021	63.56	1.52	9.23	26.23	12.55	0.00	92.55	64.96	1410	4000
10/12/2021	64.27	1.87	8.91	26.54	11.63	0.00	95.27	63.64	1303	3525
11/12/2021	66.90	1.07	8.77	26.55	11.75	0.00	100.77	64.18	1361	3618
12/12/2021	67.05	0.95	8.65	26.27	11.71	0.00	92.32	57.21	1342	4203
13/12/2021	65.56	1.07	8.77	25.83	11.19	0.00	86.73	53.29	1291	4193
14/12/2021	61.90	1.61	8.74	25.26	10.07	0.00	115.91	76.29	1057	4079
15/12/2021	59.47	2.26	8.96	26.11	8.94	0.00	119.27	82.00	1187	3787
16/12/2021	62.57	1.63	8.89	26.88	8.92	0.00	113.55	83.39	1247	3398
17/12/2021	64.41	0.94	8.42	28.16	8.99	0.00	99.14	72.96	1023	2862
18/12/2021	64.38	0.77	8.21	27.28	10.07	0.00	81.77	61.56	1003	3370
19/12/2021	65.39	0.78	7.91	24.60	11.47	0.00	76.27	55.54	966	3684
20/12/2021	67.28	0.74	7.99	24.28	10.97	0.00	102.55	80.64	914	2476
21/12/2021	71.59	0.79	7.69	24.86	10.36	0.00	122.91	100.07	910	2532
22/12/2021	73.80	1.01	8.33	25.56	9.38	0.00	132.55	110.70	871	2940
23/12/2021	73.50	1.09	8.88	26.58	9.00	0.00	128.68	103.04	870	2671
24/12/2021	66.35	1.60	9.32	27.16	8.93	0.34	122.36	88.93	821	2766
25/12/2021	63.22	1.78	9.48	27.44	10.07	0.59	85.36	51.75	831	2532
26/12/2021	63.62	1.64	9.37	27.24	11.72	1.91	77.32	45.25	842	2437
27/12/2021	66.17	1.69	9.60	27.76	12.35	0.11	90.23	57.68	850	2305
28/12/2021	65.15	1.73	9.32	27.21	12.03	0.00	66.91	44.18	774	2575
29/12/2021	65.30	1.61	9.21	27.48	12.04	0.00	72.05	43.71	714	3037
30/12/2021	65.10	1.58	9.28	26.74	13.31	0.00	71.00	41.61	943	3111
31/12/2021	66.15	1.41	9.25	26.20	13.29	0.00	65.73	44.11	900	3100
